# Beyond the Genetic Pathways, Flowering Regulation Complexity in *Arabidopsis thaliana*

**DOI:** 10.3390/ijms22115716

**Published:** 2021-05-27

**Authors:** Stella Quiroz, Juan Carlos Yustis, Elva C. Chávez-Hernández, Tania Martínez, Maria de la Paz Sanchez, Adriana Garay-Arroyo, Elena R. Álvarez-Buylla, Berenice García-Ponce

**Affiliations:** Departamento de Ecología Funcional, Instituto de Ecología, Universidad Nacional Autónoma de México, Circuito ext. Ciudad Universitaria, Coyoacán 04510, Mexico; stella.23.12@ciencias.unam.mx (S.Q.); jcyustis@ciencias.unam.mx (J.C.Y.); ec.chavez.hdz@gmail.com (E.C.C.-H.); tania.martinez@ecologia.unam.mx (T.M.); mpsanchez@iecologia.unam.mx (M.d.l.P.S.); agaray@iecologia.unam.mx (A.G.-A.); elenabuylla@protonmail.com (E.R.Á.-B.)

**Keywords:** flowering transition, genetic regulatory network, multilevel regulation

## Abstract

Flowering is one of the most critical developmental transitions in plants’ life. The irreversible change from the vegetative to the reproductive stage is strictly controlled to ensure the progeny’s success. In *Arabidopsis thaliana*, seven flowering genetic pathways have been described under specific growth conditions. However, the evidence condensed here suggest that these pathways are tightly interconnected in a complex multilevel regulatory network. In this review, we pursue an integrative approach emphasizing the molecular interactions among the flowering regulatory network components. We also consider that the same regulatory network prevents or induces flowering phase change in response to internal cues modulated by environmental signals. In this sense, we describe how during the vegetative phase of development it is essential to prevent the expression of flowering promoting genes until they are required. Then, we mention flowering regulation under suboptimal growing temperatures, such as those in autumn and winter. We next expose the requirement of endogenous signals in flowering, and finally, the acceleration of this transition by long-day photoperiod and temperature rise signals allowing *A. thaliana* to bloom in spring and summer seasons. With this approach, we aim to provide an initial systemic view to help the reader integrate this complex developmental process.

## 1. Introduction

Flowering transition is a fundamental trait in plant development that marks the end of the vegetative phase and the beginning of the reproductive state. During this process, in *Arabidopsis thaliana* (hereafter *Arabidopsis*), a species from the Brassicaceae family, the Shoot Apical Meristem (SAM) becomes an Inflorescence Meristem (IM), which develops the Floral Meristems (FMs) at its flanks. [1,2]. Then, the FMs differentiate into the flower organs [3].

The integration of developmental and physiological cues and the response to environmental signals forecast the best time for flowering to ensure the success in reproduction and the offspring’s viability [4]. In order to find the components implicated in bolting time, forward genetic analyses were performed in which *Arabidopsis* flowering mutant plants were selected under contrasting environments. Historically, four types of late-flowering mutants were found when they grew specifically under long-day (LD) or short-day (SD) photoperiods; those whose bolting time was delayed after vernalization treatment and a fourth group that showed a late-flowering phenotype under both photoperiods, but could be recovered by vernalization treatment [5]. After positional cloning, sequencing, and epistatic analyses, several factors that participate in those processes were uncovered, and they were separated into genetic pathways: the LD photoperiod pathway [6,7,8]; the late-flowering mutants under SD, resulted in plants affected in biosynthesis or signaling of gibberellins (GA); thus it was named the GA pathway [9,10,11]; the vernalization pathway [12,13,14], and the fourth group constituted the autonomous pathway [15,16].

Afterward, many other genes have been discovered to function in flowering transition by reverse genetics and expression profiling [17]. Following the former nomenclature, three additional pathways were proposed: the thermosensory pathway includes mutant plants that showed a different flowering time than wild type when grown in suboptimal temperatures [18,19,20,21]. Mutants related to the aging pathway [22,23] are predominantly late-flowering under SD conditions; and transgenics with reduced levels of trehalose 6-phosphate synthase (TPS) are late-flowering irrespective of day length [24].

It has been considered that different pathways converge into a few transcription factors (TFs) known as integrators of flowering time: FLOWERING LOCUS C (FLC), FLOWERING LOCUS T (FT); SUPPRESSOR OF OVER EXPRESSION OF CONSTANS (SOC1) and, LEAFY (LFY) [25,26,27,28,29,30]. *LFY* is one of the first genes induced at the primordial cells committed to forming the FM [31], and it is essential for floral developmental progression. Furthermore, LFY, together with APETALA 1 (AP1) and CAULIFLOWER (CAL), give the identity to the FM [32], while SOC1, AGAMOUS-LIKE 24 (AGL24), and SHORT VEGETATIVE PHASE (SVP) help to maintain this identity in the first two stages of development [33,34].

This hierarchical scheme of flowering transition has changed over time as more genes have been uncovered. The current model proposes a complex genetic network of about 300 genes that underlies floral transition [17,35,36]. In this review, we update the information about the prevention and induction of flowering transition, and more importantly, we emphasize the intricate multi-level interactions of this complex regulatory network to facilitate the integration of the mechanisms involved (sometimes simultaneously) in *Arabidopsis* reproductive phase change. However, information regarding the individual flowering genetic pathways can be consulted in different reviews [8,37,38,39].

## 2. Regulation of Flowering Repressors for Reproductive Success

Premature reproduction under suboptimal environmental conditions or at the early stages of development has implications on pollination and seed formation, with repercussions on the species’ fitness [40]. Therefore, flowering repressors are as relevant as promoter factors since they maintain the vegetative phase until specific signals trigger the reproductive transition. In *Arabidopsis*, these repressors were identified from early flowering loss of function mutants or late flowering overexpressor mutants [12,16,41,42,43,44,45,46,47].

One of these flowering repressors is *FLC* [12,42], a MADS-box transcription factor (TF) expressed during the embryonic and vegetative phase of development in which it regulates other processes too [48,49,50].

FLC negatively regulates important flowering promoters. In the leaves, it directly represses *SOC1* (a MADS-box gene) and *FT*, while in the IM, it directly delays the expression of *SOC1* and *FD* [51,52]. Conversely, the FT-FD complex directly represses *FLC* expression. This feedback loop is relevant for both flowering time and seed dormancy [50,53].

FLC and other members of this clade, such as FLOWERING LOCUS M (FLM/MAF1), MADS AFFECTING FLOWERING 2 to 5 (MAF2-5), and SVP, have functional redundancy, partly because of their interchangeable protein protein interactions [21,54,55,56,57]. The combinatorial activities of these MADS-domain proteins fine-tune the expression of *SOC1* and *FT* in response to temperature changes, vernalization, and photoperiod [21,54,55,58,59,60,61].

Global binding mapping of FLC and SVP to their target loci in mutant backgrounds has shed light on how these MADS-domain proteins act as flowering repressors [48,62,63]. A remarkable example indicates that FLC and SVP can bind to their targets together as a dimer, redundantly (both of them are able to bind to the same regulatory sequences), or independently (they bind to different sites and additively represses gene expression). Using this analysis was found that SVP-FLC binds exclusively as a complex to genes, such as *TEMPRANILLO1* (*TEM1*) and *CONSTANS-LIKE 1* and *4* (COL1/4), while SVP targets *SEPALLATA3* (*SEP3*) and *SCHLAFMÜTZE* (*SMZ*) without the requirement of FLC. Furthermore, both proteins can bind independently to *FT* and *SOC1* genes [56]. Interestingly, FLC and SVP regulate GA metabolism in a complex manner. On one hand, SVP upregulates GA2-oxidases (*GA2OX*) genes that encode GA-catabolic enzymes, and both FLC and SVP repress *GA20OX2*, a key GA_4_ biosynthetic enzyme. Consistent with this, single and double *svp flc* mutants are early-flowering, and the *svp* plants contain elevated GA levels [56,64]. On the other hand, the SVP-FLC complex represses *GA2OX* genes [56], but its implication in flowering transition needs further investigation.

Another genome-wide study among FLC, SVP, and SOC1 showed that they share common targets, but SOC1 function in an antagonistic manner [62]. FLC and/or SVP recruit(s) the Polycomb repressive complex (PRC) to *TARGET OF FLC AND SVP 1* (*TFS1*) gene to deposit the trimethylation of histone H3 lysine 27 (H3K27me3), which is a repressive epigenetic mark. *TFS1* encodes a B3-type TF, and *tfs1* plants are late flowering, particularly under short days. After *FLC* is silenced, SOC1 acts as a pioneer TF that associates with the histone demethylase RELATIVE OF EARLY FLOWERING 6 (REF6) and the chromatin remodeler BRAHMA (AtBRM), allowing the binding of the SQUAMOSA BINDING PROTEIN LIKE 9 (SPL9) TF to the *TFS1* locus. Moreover, the RNA-polymerase II (RNAPII) is recruited through the Mediator (MED) complex, resulting in the upregulation of *TFS1* at the shoot apex [65].

Interestingly, brassinosteroids (BRs) also inhibit floral transition and promote vegetative growth in two ways. In one of them, BRs induces the expression of *FLC, FLM, MAF4*, and *MAF5* through BRASSINAZOLE-RESISTANT 1 (BZR1), which in the case of *FLC* recruits EARLY FLOWERING 6 (ELF6), that removes H3K27me3 repressive marks. The other way is by direct BZR1 repression of *SOC1* [66].

When plants reach a developmental stage for flowering competence, *FLC* must be silenced. Multiple proteins participate in *FLC* transcriptional and posttranscriptional regulation. Although our knowledge is still limited, the chromatin remodeling proteins, such as the histone demethylase FLOWERING LOCUS D (FLD); the HISTONE DEACETYLASES 5 and 6 (HDA5, HDA6) together with FVE (or MULTICOPY SUPPRESSOR OF IRA 4; MSI4), could be associated in a co-repressor complex at the *FLC* locus [67,68,69,70]. Moreover, LUMINIDEPENDENS (LD; homeodomain protein), DOMAINS REARRANGED METHYLTRANSFERASE 2 (DRM2; DNA methylation), PROTEIN ARGININE METHYLTRANSFERASE 5 and 10 (PRMT5, PRMT10), and REF6 (H3K27me3 histone demethylase) are also important for *FLC* epigenetic silencing [71,72,73,74,75,76].

Besides chromatin modifiers, there is a group of proteins that negatively affect *FLC* mRNA processing: FCA, FPA, and FLOWERING LOCUS KH DOMAIN (FLK) are RNA-binding proteins [77,78,79]; FY, is an RNA 3′-end processing factor [80], and PCF11P-SIMILAR PROTEIN 4 (PCFS4) participates in alternative polyadenylation [15,37,69,81,82,83]. However, not all KH-domain RNA-binding proteins inhibit *FLC* transcript accumulation. Some members of the HUA PEPPER (PEP) family promote its messenger stabilization [84,85,86].

Apart from repressing *FLC*, FCA and FVE may also act as negative regulators of *SVP*, since the late-flowering phenotype of *fca-9* and *fve-3* was suppressed by mutations in the *SVP* gene [21]. Alternatively, *fca-9* and *fve-3* may be disrupting the *FLC*-dependent functions of *SVP* in flowering.

Far from being a linear pathway, developmental downregulation of *FLC* expression includes various complex molecular processes in which both chromatin modifiers and RNA-binding proteins participate [15,69,74]. There are two possibilities to explain the interdependence of these two groups of proteins. The first one considered a set of antisense long noncoding RNAs (lncRNAs) collectively called *COLD INDUCED LONG ANTISENSE INTRAGENIC RNAs* (*COOLAIR*), which are transcribed from the 3’-end of *FLC* and proximately polyadenylated by FCA, FPA, and FY, and spliced by PRP8 [87]. Some of the *COOLAIR* variants could lead to FLD recruitment, which demethylates H3K4me2, reducing the transcription of both sense and antisense *FLC* mRNAs [83,88]. Alternatively, there is evidence that FCA binds to CURLY LEAF (CLF), a PRC2 subunit, in the presence of *COOLAIR* for H3K27me3 deposition at the *FLC* locus [89].

Furthermore, Casein kinase 2 (CK2) phosphorylation and phosphatase 2A (PP2A) dephosphorylation may regulate *FLC* expression and protein stability or activity. In the *ck2 α*-subunit triple mutant, non-phosphorylated components of the autonomous pathway led to higher *FLC* levels and, consequently, a late flowering phenotype [90]. Meanwhile, PP2A acts as a positive or negative regulator of *FLC* expression, depending on the B subunit of the PP2A trimeric complex [91]. At the posttranslational level, phosphorylation modifies the flowering repression activity mediated by FLC. In this regard, transgenic plants carrying a *FLC-FLAG* construct that mimics FLC in the phosphorylated state are early flowering [92]. SUMOylation stabilizes the FLC protein, and a mutation in this site overrides FLC repressor activity [93]. Moreover, the ubiquitination of FLC by the E3 ubiquitin ligase activity of SINAT5 may reduce FLC levels, promoting flowering transition [93].

*FLM* and *SVP* are also regulated at the posttranscriptional and posttranslational levels [60,61,94]. LATE ELONGATED HYPOCOTYL (LHY) and CIRCADIAN CLOCK–ASSOCIATED 1 (CCA1), two circadian clock proteins, accelerate flowering in part by reducing SVP protein abundance by targeting it for degradation at dawn [54]. Additionally, SVP activity is modulated by DNAJ HOMOLOG 3 (J3), a J-domain chaperone that interacts directly with SVP in the nucleus and prevents SVP binding to *SOC1* and *FT* regulatory sequences. *J3* is expressed throughout Development but it is upregulated by long-day photoperiod, gibberellins, and vernalization in a *FLC* independent manner [95].

During the vegetative phase, SVP upregulates *APETALA 2* (*AP2*)-like genes directly or indirectly via transcriptional repression of the *microRNA 172A* (*MIR172A*) [62,96]. Meanwhile AP2-like TFs negatively regulate each other in a complex network of transcriptional interactions [47,97]. AP2 and SMZ directly bind to *SOC1* and *FT* promoters and repress their expression [47,97]. Thus, downregulation of *AP2-*like genes by either GIGANTEA (GI) or developmental induction of miR172, promote floral transition [98,99]. Interestingly, plants overexpressing SMZ had a late-flowering phenotype which was independent of *FLC* and *SVP*, but it was completely suppressed in the *flm* mutant [47]. The latter is partly explained because *FT* expression was restored [47]. Therefore, *SMZ* repression depends strictly on *FLM*, but the molecular mechanism is still unknown.

The *TEM1* and *TEM2* genes are also part of the *AP2*-like family of TFs, but miR172 does not downregulate them. TEM1 and TEM2 repress the transcription of *FT* [99], *MIR172* genes [100], and the GA biosynthetic genes *GA3OX1* and *GA3OX2* [101], hence inhibiting flowering. GI is a direct positive regulator of *FT* transcription under SD conditions [102] and a direct inducer of *CO* in LD photoperiod [103]. Interestingly, when GI interacts with TEM1, TEM2, and SVP, it probably interferes in their repression activity over *FT* in SD. However, GI transcription in this condition is also limited by FAR 1 RELATED SEQUENCE 7 (FRS7) and FRS12 TFs [104]. Moreover, the quantitative balance between CO and TEM1/2 determines *FT* levels in LD [99].

AGAMOUS-LIKE 15 (AGL15) and AGL18 are other flowering repressors belonging to the MADS-box TFs family [46]. Both control flowering time by repressing *FT* and *SOC1* [46,105] and inducing *MIR156* [106]. AGL15 also possibly regulates other genes such as *FLC, SVP, MAF3, MAF5, AGL18, MIR172, SPL3, AGL19, AGL24, FRUITFULL* (*FUL*), and *LFY* [105,106,107]. The *agl15 agl18* double mutant showed an additive effect on flowering time acceleration when it was crossed with *flc, flm*, and *svp* mutants, indicating that they act independently [46]. Interestingly, there is a large percentage of overlap between SVP and AGL15 targets, including *AP2* and the *AP2*-like genes *TEM1, TARGET OF* *EARLY ACTIVATION TAGGED 1* (*TOE1*), and *TOE3*, as well as *MIR172A, SOC1,* and *SPL11* [62,105,107]. AGL15/18 may repress flowering during embryonic Development young seedlings, or under specific growth conditions, while SVP repressor activity affects later stages of vegetative development [46,105].

The repressed state of *SOC1* and *FT* before flowering partially depends on the interaction of PRC2 components CLF, EMBRYONIC FLOWER 2 (EMF2) and MSI1 with the catalytic subunit of DNA Polymerase Ɛ, EARLY IN SHORT DAYS 7 (ESD7) [108,109]. The LIKE HETEROCHROMATIN PROTEIN 1 (LHP1), also known as TERMINAL FLOWER 2 (TFL2) [110], acts in repressing euchromatic genes [111]. LHP1 recognizes the H3K27me3 enriched regions of *FLC, FT*, and *SOC1* genes [112,113,114,115] and it can also interact with MSI1 and ESD7, linking PRC2 and the replication machinery to maintain high levels of H3K27me3 at a particular locus [109,111,116]. Repression by LHP1 requires the DNA polymerase α catalytic subunit INCURVATA 2 (ICU2) [111]. Hence, the association between PRC2 and the DNA replication machinery could be important for epigenetic memory after DNA replication, particularly during seed formation [116].

EARLY BOLTING IN SHORT DAYS (EBS) and its paralog SHORT LIFE (SHL) are required for *FT* and *SOC1* repression, respectively [117,118]. They have a bivalent bromo-adjacent homology (BAH) domain that recognizes the H3K27me3 mark and a plant-homeodomain (PHD) motif that binds to H3K4me3 [119]. EBS and SHL repression function requires the interaction with HDA6 [118] and EMBRYONIC FLOWER 1 (EMF1) performing as a PRC1-like complex that reads the H3K27me3 mark introduced by the PRC2 complex to silence gene expression [120]. Contrary to direct repression of *FT* and *SOC1* by EBS and SHL, the SIN3 LIKE (SNL) proteins function as a scaffold for histone deacetylase assembly complexes to repress TOE1, TOE2, and SMZ, indirectly allowing *FT* expression in LD growing plants [121].

Recently, another epigenetic component was unraveled from a mutagenized quintuple mutant (*svp-41 flc-3 ft-10 tsf-1 soc1-2*) screening [122]. CHROMATIN REMODELING 4 (CHR4) is a PICKLE (PKL) homolog that affects H3K27me3 and H3K4me3 levels at a subset of loci in the genome. One of these loci, *SPL15,* was highly enriched with the H3K4me3 mark in the *chr4-2* mutant compared to wild-type, correlating with higher transcript levels of this gene. It seems that CHR4 interacts with many TFs and other chromatin remodelers in protein complexes that differentially affect the floral transition [122].

Another important mechanism involved in vegetative phase maintenance consists on the negative posttranscriptional control of miR156 over *SPL* transcription factors [22,123,124,125]. Interestingly, aging promotes downregulation of *pri-MIR156* transcription by increasing H3K27me3 marks at the *MIR156A* and *MIR156C* loci [125]. PRC2 complex containing CLF, SWINGER (SWN), and the chromatin remodeler PKL participates in this developmental repression [125]. On the contrary, AtBRM acts antagonistically to SWN, promoting *MIR156A* expression during the early vegetative stage [126]. Transcription from *MIR156A/C* is also negatively regulated by sugars [127,128] and by the Mediator CDK8 subunits CENTER CITY (CCT/MED12) and GRAND CENTRAL (GCT/MED13), which can repress *MIR156A/C* independently, but they have a more substantial effect when acting together [129,130]. Moreover, CCT and GCT promote flowering transition by repressing *FLC* expression [129]. Intricate feedback loops affect developmental phase change linking it to flowering competence. After miR156 levels decrease, SPL9 and SPL10 upregulate the expression of *MIR172B*. Additionally, miR172 abundance is also regulated by GI in response to LD photoperiod [98] and by FCA during thermal regulation [131]. In return, miR172 negatively regulates the flowering repressors *AP2* and *AP2*-like genes *SMZ, SCHNARCHZAPFEN* (*SNZ*)*, TOE1, 2*, and *3* via translational inhibition [47,132].

In conclusion, mutual inhibition between flowering repressors and promoters, in collaboration with epigenetic modifiers, controls the precise moment of flowering transition.

## 3. Effect of Suboptimal Temperature and Vernalization in Flowering Time

Among the multitude of environmental signals to which plants respond to synchronize their development to adequate conditions, low temperatures affect flowering time in several plant species [133,134]. In *Arabidopsis*, temperatures between 5–16 °C, negatively affect flowering in many natural accessions [135]. On the contrary, vernalization accelerates flowering in plants that undergo prolonged periods of low temperatures (4 °C or lower) and afterward reach optimal growth temperatures in spring and summer seasons [136].

The molecular mechanisms that regulate plants’ response to subtle changes in ambient temperature have begun to be discovered [137,138]. Through a phylogenetic footprinting approach, it was found that FLM is relevant on the control of flowering in *Arabidopsis* natural populations in response to ambient temperature changes. This regulation resides within the noncoding regulatory regions that affect the expression of *FLM* [135].

Furthermore, *FLM* is subject to temperature-dependent alternative splicing that results in the inclusion of the second (FLM-β) or the third exon (FLM-δ), respectively [60,61]. Interestingly, at lower temperatures the *Arabidopsis* SPLICING FACTOR 1 (AtSF1) preferentially binds to the first intron branch site of *FLM* pre-mRNA producing the flowering-repressive FLM-β isoform. Accordingly, the *atsf1-2* mutant which has very low levels of FLM-β and significant higher levels of FLM-δ, is early flowering, but it has lost the sensitivity to temperature control [139].

Between 10 °C and 16 °C, SVP associates with the FLM-β isoform and represses *SOC1, FT*, and *TWIN SISTER OF FT* (*TSF*) expression and promotes *TEM2* transcription. Thereby, the SVP-FLM-β complex prevents precocious flowering under suboptimal temperatures [60,61]. On the contrary, it was proposed that the FLM-δ isoform may compete with FLM-β for the binding to SVP at higher temperatures (27 °C). Since the SVP-FLM-δ complex is impaired in DNA binding, it could be acting as a dominant-negative flowering repressor [60,61]. After deleting specifically the second or third exons by CRISPR/Cas9 technology, plants expressing only the FLM-β were late flowering, as expected. Meanwhile, plants with the FLM-δ isoform showed an early flowering phenotype, but not as early as the *flm-3* loss-of-function mutant, which would be expected if FLM-δ acts as a dominant-negative [140]. Hence, it seems the net reduction in the abundance of FLM-β at optimal temperatures is sufficient to disrupt the formation of the repressive complex with SVP [61,140,141]. In this sense, *FLM* transcript levels are reduced through alternative splicing coupled with nonsense-mediated mRNA decay (AS-NMD), resulting in a net loss of SVP-FLM-β complex [94]. Furthermore, SVP protein degradation contributes to reducing the SVP-FLM-β heterodimer levels at high temperatures [60].

As FLM, MAF2 prevents flowering after short periods of cold, and mutations in *MAF* genes decrease plants’ sensitivity to temperature changes [59,142]. *MAF2* undergoes a temperature-dependent alternative splicing process too [143,144]. At 16 °C, the predominant MAF2var1 isoform interacts with SVP to repress flowering, whereas at 27 °C, the MAF2var2 isoform accumulates. The latter isoform cannot interact with SVP, and its transcription hinders the accumulation of *MAF2var1* [144].

There are several miRNAs differentially expressed at 16 °C and 23 °C. From those involved in temperature-dependent regulation of flowering, miR156 and miR169 are upregulated at 16 °C, while miR172 accumulates at 23 °C. Interestingly, accumulation of miR172 depends posttranscriptionally on FCA which is preferentially accumulated at optimal temperatures [131]. *FCA* itself is regulated by alternative splicing and only the *FCA*-γ mRNA variant produces a functional protein [145,146]. Conversely, target genes of those microRNAs showed an anti-correlative accumulation [147]. Among them, cleavage of *SPL3* mRNA by miR156 is enhanced at 16 °C. Downregulation of SPL3, results in lowering *FT* expression, which is a direct target of SPL3 in the leaves, preventing flowering under suboptimal temperatures [148].

Mutants in *cryptochrome 1* and *2* genes (*cry1* and *cry2*) in combination with *phytochrome A* (*phyA*) show a drastic flowering delay under 16 °C compared to 23 °C [18]. Also, *phyB* single mutant and the *phyA phyB phyD* triple mutant are early flowering at optimal temperatures, but they flower at the same time as wild-type plants at 16 °C. In this case, phyE which is still active in the triple mutant, mediates this response by indirectly repressing *FT* expression under cooler temperatures and this process occurs independently of FLC and FLM mediation [19]. Therefore, it has been suggested that phyB and other photoreceptors function as thermoreceptors [149].

The *constitutive photomorphogenic 1* (*cop1*) mutant has an early flowering phenotype that shows almost no delay response to low temperatures. At 16 °C, COP1, a RING-finger E3 ubiquitin ligase, is stabilized and favors GI turnover. Again, this condition abolishes *FT* direct induction by GI, suppressing in this way flowering induction [150]. Therefore, posttranslational regulation is also important in the flowering response to suboptimal temperature changes. Additionally, COP1 is also important in CO turnover in response to photoperiod (see the corresponding section).

In brief, low temperature triggers different mechanisms that prevent *Arabidopsis* plants to flower in autumn or during subtle climate changes.

Vernalization requirement on the other hand, varies significantly among different plants and even between different natural populations of the same species [151]. Studies on the genetic source of natural variation showed that the vernalization requirement in winter-annual *Arabidopsis* accessions depends on *FRIGIDA* (*FRI*) and, or *FLC* alleles [136].

Before winter, the *FLC* locus is enriched with chromatin marks related to transcriptional activation, such as H3K4me3, H3K36me3, and histone acetylation, deposited by a FRI-supercomplex in plants with an active *FRI* allele [152,153]. However, in the absence of a functional FRI, other members of the FRI-complex (FRI-C) maintained basal levels of *FLC* in summer-annual accessions [154].

**Figure 1:**
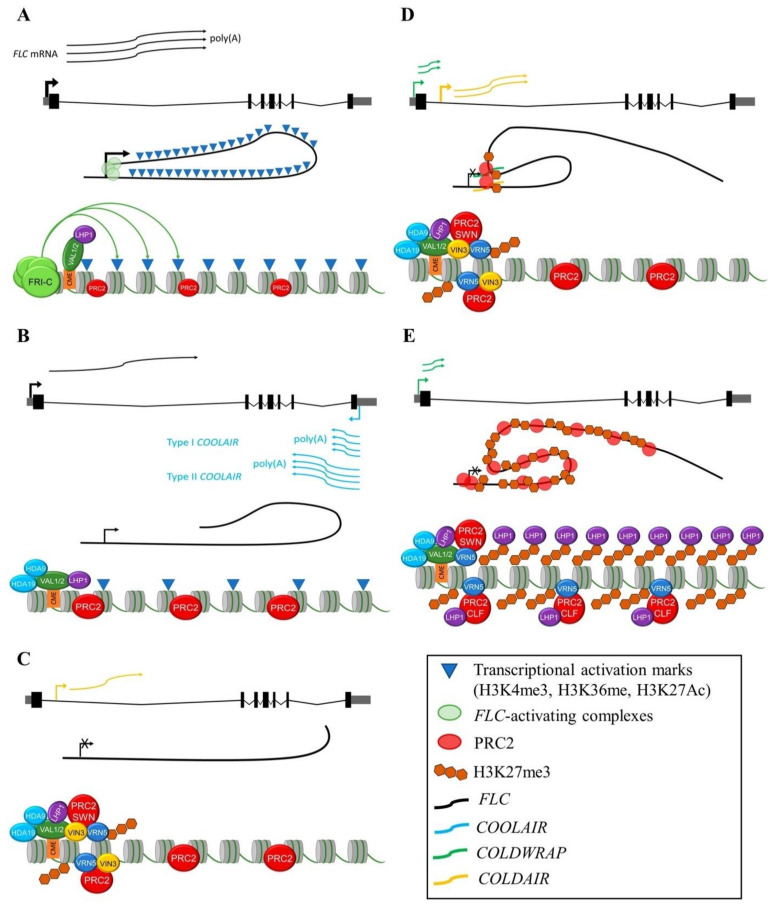
*FLC* silencing events during vernalization. Epigenetic factors and non-coding RNAs carry out this process. (**A**) Before vernalization, *FLC* is transcriptionally active. (**B**) Initial *FLC* silencing by cold-induced *COOLAIR* (type I). (**C**) The PHD dimer VIN3 and VRN5 binds to the PRC2 complex increasing the deposition of the H3K27me3 mark at the nucleation region. (**D**) After prolonged exposure to cold, *COLDAIR* and *COLDWRAP* in association with PRC2 promote the formation of a repressive chromatin loop reinforcing *FLC* silencing. (**E**) Spreading of the H3K27me3 mark by CLF is maintained by LHP1 after plants returned to warm temperatures. A scheme of the *FLC* gene and the mRNAs produced (top), the *FLC* DNA structure (middle), and some of the proteins participating in nucleosome modifications (bottom) are shown in each panel.

DNA structural conformations at the *FLC* locus are important for its own transcriptional regulation (Figure 1A). For example, a DNA loop formed between the 5′- and 3′-flanking regions of the *FLC* locus is disrupted at the beginning of vernalization, switching the chromatin conformation from an active to a repressive state [155]. SWITCH/SUCROSE NONFERMENTING (SWI/SNF) ASSOCIATED PROTEIN 73B (SWP73B)/BAP60 could be participating in the release of this *FLC* DNA loop as an early step in *FLC* repression [156]. The formation of an R-loop at a heterochromatic region of the *COOLAIR* promoter, stabilized by the homeodomain protein *Arabidopsis thaliana* NODULIN HOMEOBOX (AtNDX), inhibits *COOLAIR* expression and consequently promotes *FLC* expression [157]. However, the accumulation of *COOLAIR* at the beginning of vernalization treatment, correlates with drastic reduction in the levels of the encoding *FLC* transcript, independently of PRC2 [87,158,159,160], suggesting there is a mechanism that allows transcription of only one DNA strand at the time [161] (Figure 1B).

A dynamic change in the chromatin environment is required for *FLC* silencing in which *COOLAIR* promotes the cold-induced reduction of H3K36me3 and H3K4me3 and the increase of the H3K27me3 repression mark particularly at the nucleation region [158,162]. This region corresponds to the first exon and the beginning of the first intron encompassing three nucleosomes and includes a 47 bp cis-regulatory element with two identical RY motifs named the Cold Memory Element (CME). VP1/ABI3-LIKE 1 (VAL1) and VAL2 proteins directly bind to the CME and recruit two histone deacetylase proteins HDA9 and HDA19. VAL1 also associates with the apoptosis and spliceosome (ASAP) complex and LHP1 [163,164,165].

PRC2 whose components include VERNALIZATION 2 (VRN2), FERTILIZATION-INDEPENDENT ENDOSPERM (FIE), MSI1 and predominantly the SWN methyltransferase initially deposits the H3K27me3 mark at the nucleation region [111,166,167]. To accomplish this, cold-induced VERNALIZATION INSENSITIVE 3 (VIN3) heterodimerizes with VERNALIZATION 5 (VRN5), two PHD family members [166]. These PRC2 accessory proteins also interact with MSI1 and VAL1/2, linking PRC2 to the nucleation region [163,164]. VIN3 induction by NAC with TRANSMEMBRANE MOTIF 1-LIKE 8 (NTL8) is essential for the plant to sense the difference between short and prolonged periods of cold [168,169,170] (Figure 1C).

Later during vernalization, two other lncRNAs are transcribed from the *FLC* locus in the sense direction: *COLD ASSISTED INTRONIC NONCODING RNA* (*COLDAIR*) is transcribed from the first intron [171], and *COLD OF WINTER-INDUCED NONCODING RNA FROM THE PROMOTER* (*COLDWRAP*) from the proximal *FLC* promoter. *COLDAIR* and *COLDWRAP* accumulation levels, peak at 20 and 40 days, respectively, after vernalization initiation [159]. Interestingly, both of them associate with the PRC2 complex to promote the formation of a repressive chromatin loop between the sites of transcription initiation of these two noncoding RNAs, contributing to the repressive state of *FLC* [159] (Figure 1D).

Once plants return to warmer temperatures, the PHD-PRC2 complex without VIN3 (whose expression declines during vernalization) spreads the H3K27me3 mark throughout the entire *FLC* gene [166]. LHP1 together with CLF methyltransferase, are required to maintain the *FLC* epigenetic silencing [112,113]. Consistent with this, both proteins bind to different components of the replication machinery probably to methylate newly deposited histones [109,161] (Figure 1E).

The epigenetic regulation of *FLC* has become an invaluable model to explain flowering in response to vernalization in *Arabidopsis* [172]. However, there is little information about the vernalization process and the gene network underlying flowering regulation in response to this seasonal condition. In this sense, a recent transcriptomic and epigenomic analysis showed differential expression of genes that may complement this knowledge gap [173]. Moreover, there are still questions to be resolved related to the promotion of flowering after vernalization. For example, *FLC* silencing by vernalization is necessary but not sufficient for *SOC1* expression, suggesting that positive regulators are also required [27,28]. Furthermore, non-vernalized C24 accession plants have increased *SOC1* mRNA levels around the flowering time, even though there is no decrease in *FLC* expression yet [174]. Thus, it is possible there are different inductive mechanism that bypass FLC repressive activity, or FLC activity is inhibited by posttranslational regulation.

Apart from FLC, AGAMOUS-LIKE 19 (AGL19) and AGL24 are the only TFs known to participate in flowering transition in response to vernalization. Both of them, are induced by vernalization, independently of *FLC* silencing, and *agl19* and *agl24* mutants are late-flowering compared to wild-type plants after vernalization [175,176].

*AGL19* and *AGL24* are repressed by HDA9 under short-day conditions, probably to avoid early flowering [165,177,178] and *AGL19* is epigenetically silenced by the EMF-2 Polycomb repressive complex in non-vernalized plants [179]. However, after a vernalization, *AGL19* is induced when the H3K27me3 marks are reduced, particularly at the 5′ region of the first intron [176].

It is known that AGL24 participates in flowering transition in response to other signals [180,181]. However, currently, there is no information about AGL19 and AGL24 regulatory functions in response to vernalization nor their genetic relationships. Further research is required to establish their role in this process.

## 4. The Role of Endogenous Cues in Flowering Regulation

Plant’s endogenous cues that participate in flowering transition are present independently of the season, although environmental signals influence them. In this section, we describe the essential role of gibberellins and the effect of the trehalose-6-phosphate signaling, which constitutes a sensor of sugars availability and hence the plant’s reserves to support reproduction [24,182,183]. Both signals are interconnected with the miR156-SPLs-miR172 regulatory module (or the aging pathway) [184,185] and the MADS-domain TFs to induce flowering transition.

Two microRNAs, miR156 and miR172, show opposite temporal expression patterns and functions and have an essential role in phase transitions during plants’ development. miR156 is expressed during the early stages of development and is involved in juvenile phase maintenance, while miR172 levels increase with aging and promote adult vegetative traits, as well as the transition to the reproductive stage [22,123,186,187]. Ten members of the *Arabidopsis SPL* TFs family are posttranscriptionally repressed by miR156 [188]; from these, SPL2, SPL3, SPL4, SPL5, SPL9, SPL10, SPL11, and SPL15 have been implicated in flowering transition [39,189,190,191,192,193].

As the plant ages, miR156 levels gradually decrease, allowing *SPL* mRNAs accumulation. In turn, SPL9, SPL10, and SPL15 induce reproductive transition by regulating genes related to flowering and binding to the *MIR172B* promoter [123,191]. Subsequently, the accumulation of miR172 indirectly promotes flowering transition by targeting *AP2* and *AP2*-like flowering repressors, which repress *SPL3*, *SPL4*, and *SPL5* (*SPL3/4/5*) genes [123,187,194]. Also, SMZ suppresses *SOC1* and *AP1* genes [47].

SPL10 and its closest homologs SPL11 and SPL2 have been implicated in flowering regulation as well [192,193]. Chromatin immunoprecipitation (ChIP) experiments with SPL10 showed that *FUL* and *AP1* are their direct targets. Meanwhile, MED25 enrichment in *FUL* and *AP1* promoters is severely reduced in the triple mutant *spl10 spl11 spl2*, indicating that these SPLs are required for MED25 recruitment to these loci [193].

Interestingly, two TFs involved in phyA signaling, FAR-RED ELONGATED HYPOCOTYL 3 (FHY3) and FAR-RED IMPAIRED RESPONSE 1 (FAR1), have been shown to interact with SPL3/4/5. These interactions impede the latter’s binding to the promoters of *FUL, LFY, AP1*, and *MIR172C* delaying flowering transition when plants grow under white light. However, under shade conditions, FHY3 and FAR1 protein levels decrease, and SPL3/4/5 can induce those genes [195]. This mechanism probably allows plants that compete with their neighbors for light to flower earlier and ensure better survival probabilities for their descendants.

SPL impact on flowering time has been relatively difficult to study due to the high functional redundancy among their members. Single knockout mutants do not show a late flowering phenotype, while double and triple mutants such as *spl3 spl4 spl5*, *spl9 spl15*, and *spl10 spl11 spl2* show a more significant delay in flowering, particularly under SD [192,193,194]. Exceptionally, the single mutant *spl15-1* flowers extremely late compared to wild-type plants when grown in SD, suggesting that SPL15 plays a relevant role in flowering regulation under this photoperiod [191]. Indeed, SPL15 induces *FUL* and *MIR172*. These three genes show a synergistic effect on flowering promotion [196]

GA constitute a group of tetracyclic diterpene compounds that have multiple functions. It was Lang (1957) who first described the effect of these hormones as bloom inducers [197]. Exogenous treatments with GA_3_ or GA_4_ compounds accelerate flowering time in *Arabidopsis*, although GA_4_ seems to be the active molecule in flowering regulation since it is the one that accumulates the most at the shoot apex. High levels of this particular compound correlate with strong induction of *LFY* and *AP1* at the incipient FM [198].

GA signaling occurs through the degradation of DELLA proteins [199,200]. This class of GRAS-family proteins has five members in *Arabidopsis*: REPRESSOR OF *ga1-3* (RGA), GIBBERELLIN INSENSITIVE (GAI), and RGA-LIKE 1 (RGL1), RGL2, and RGL3 [201,202,203,204]. DELLAs repress the function of TFs by binding to them and interfering with their activity [205]. In this way, RGA and GAI bind to the B and C subunits of the NUCLEAR FACTOR-Y (NF-Y) heterotrimeric TF complex, which impedes its association with CO. Furthermore, DELLA proteins directly bind to the CCT domain of CO, inhibiting *FT* induction under LD conditions [206,207]. On the contrary, in the presence of GA, NF-Y mediates CO upregulation of *SOC1* partly through REF6 regulation [115].

GA signaling begins when their levels increase, and they bind to the receptor GIBBERELLIN INSENSITIVE DWARF 1 (GID1). This union induces a conformational change, allowing GID1 to interact with DELLA proteins. The formation of the GA-GID1-DELLA complex then promotes the interaction between DELLAs and the F-box protein SLEEPY1 (SLY1), which is part of the SCF^SLY1^ E3 Ubiquitin Ligase complex, leading to ubiquitination of DELLAs and, consequently, to their degradation through the proteasome 26S [208,209,210,211].

Endogenous cues regulate reproductive transition by activating flowering associated genes in the leaves and the shoot apex, but under SD conditions, signaling at the SAM becomes essential to induce flowering. An illustrative experiment showed that if DELLAs or GA catabolic enzymes are expressed either by the phloem-specific promoter *SUCROSE 2* (*SUC2*) or from the IM-specific *FD* promoter, both delay flowering in LD growing plants. However, only the lines expressed in the IM affect flowering time under SD conditions [11,212]. Thus, GA signaling in the SAM is required to induce flowering under SD photoperiod in *Arabidopsis* [213]. Consistent with this, mutants impaired in GA biosynthesis (*ga1-3* and *ga1-6*), or the *35S:miR156* line, show moderate late-flowering phenotypes when grown in LD, whereas they flower very late or even fail to do it under SD [212,214].

The basic helix-loop-helix (bHLH) family of TFs is involved in GA biosynthesis and action during the flowering transition. The mutant *no flowering in short day* (*nfl*) affects GA biosynthetic genes expression, and it can be rescued by GA addition, indicating that NFL controls flowering transition through regulation of GA biosynthesis [215]. Furthermore, GA-induced activation of bHLH48 and bHLH60 promote *FT* expression, independently of CO [216]. Moreover, under LD, GAs repress the MYC3 bHLH TF activity, which is stabilized by DELLAs and constitutes a direct repressor of *FT* in a CO antagonistic manner [217].

Induction of *SPL3/4/5* by GA in the SAM is mediated by SOC1 [11,218]. Furthermore, GAs are required to release SPL15 from RGA and GAI inhibition. SPL15 then associates with SOC1, which, in collaboration with the REF6 and MED18 complex, induce *FUL* and *MIR172B* [191]. Additionally, GAs are important for upregulation of *SPL10*, which indirectly contributes to *FT* induction via miR172 accumulation in the leaves [47,123].

Interestingly, DELLA/GA signaling affects SPL9 function by two opposing mechanisms. On one hand, GA-induced ubiquitination of RGA is required for SPL9 to induce *SOC1* [219]. On the other hand, SPL9 seems to require the presence of DELLAs to induce *AP1* transcription, since SPL9-mediated expression of *AP1* strongly decreases when plants are treated with GAs [219]. The latter mechanism indicates that DELLAs also participate as co-activators [217,219,220].

**Figure 2:**
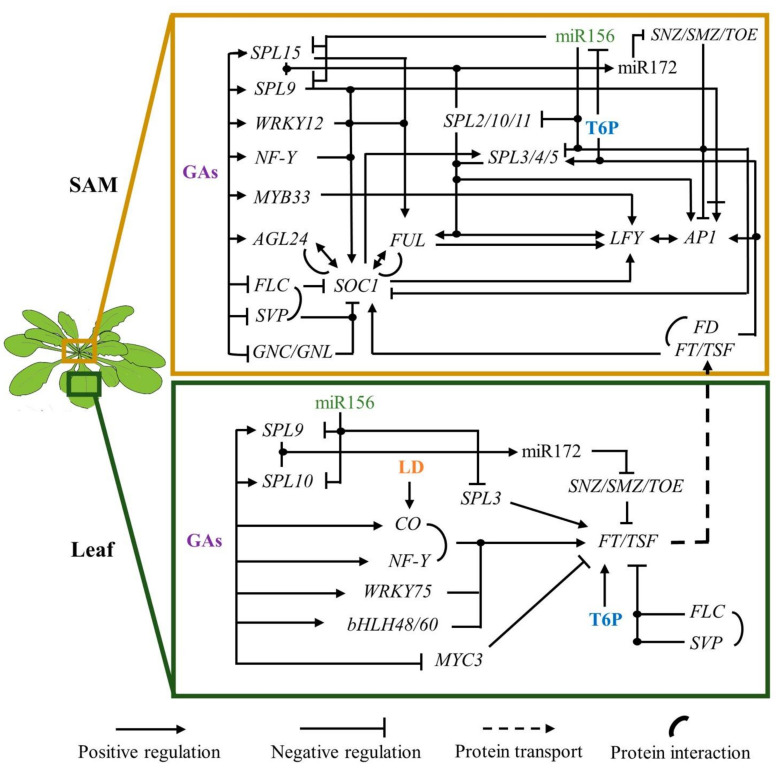
Gene regulatory network that promotes flowering transition in response to endogenous cues. Intricate regulations among the miR156-SPLs-miR172 module, and GAs and T6P signaling lead to *FT* induction in the leaves and *LFY* and *AP1* at the flanks of the IM. MADS-box, SPLs, AP2-like, and WRKY TFs have a preponderant role in flowering transition. SPL9 dual regulation on *AP1* (positive and negative bars), implies that in the presence of DELLAs, *AP1* is induced, but GA treatment drastically reduced it. Line intersections with dots indicate molecular interactions.

Besides SPLs, GA signaling contributes to flowering transition by regulating the expression and activity of some MADS-box TFs in different manners (Figure 2). For example, FLC binding to *SOC1* and *FT* regulatory regions is enhanced in the presence of RGA; thereby, its degradation by the addition of GA_3_ reduces *FLC* repressive action [220]. Moreover, GAs induce *SOC1* through the activity of NF-Y, or by downregulation of *GATA NITRATE-INDUCED, CARBON METABOLISM INVOLVED* (*GNC*), and its paralog *GNC-LIKE* (*GNL*), which encode direct repressors of *SOC1* [115,221].

Also, DELLA proteins inactivate some of the WRKY TF family members implicated in flowering. WRKY75 and WRKY71 are direct regulators of *FT*, and WRKY71 also binds to *LFY*, although it is not clear if its TF activity depends on GA signaling [222,223]. WRKY12 and WRKY13 have opposite functions in flowering. WRKY12, in association with SPL10, induces *MIR172B*, while WRKY13 and SPL10 repress it [224]. Both of them interact with GAI and RGL1, and the degradation of these DELLAs by GAs enables WRKY12 to upregulate *FUL* expression directly and *SOC1* indirectly [225]. Subsequently, FUL induces flowering partly in collaboration with SOC1 [226].

It has been proposed that FUL can also counteract FLC flowering repression by competing for the association with SVP. As said previously, FLC and SVP repress *SOC1* and *FT* [21,54,55]. However, after accumulation of FUL, SVP interacts with FUL to induce *SOC1* expression [226]. Furthermore, SOC1 and FUL suppress SVP repression over *GA20OX2*. Thus, by lowering the repressor activity of SVP, GA levels rise, reinforcing flowering transition [55,64]. SOC1 and AGL24 mutual induction is also enhanced in response to GA [227]. Finally, *XAANTAL2* (*XAL2*) is another member of the MADS-box family involved in flowering, probably in response to GA and independently of *SOC1*, since the double mutant *xal2-2 soc1-7* was unable to flower in response to GA_3_ treatment after 88 days under SD conditions [228].

GAs regulation of MADS-box genes leads to the induction of *LFY* expression [226,227,229]. However, GAs can upregulate *LFY* expression by an independent mechanism. MYB33 accumulates in the shoot apex in response to GA_4_ addition [230]. Furthermore, MYB33 binds to a highly conserved region in the *LFY* promoter. Hence, when this region is mutated, *LFY* expression is severely reduced under SD photoperiod [10].

The carbohydrates were long considered to be involved in the vegetative to reproductive transition [231,232]. Indeed, the addition of glucose or sucrose to *Arabidopsis* and other species generally accelerates flowering, although the effects may vary depending on the sugar concentration and the plant’s developmental stage [128,183,233]. The mechanisms by which sugars regulate flowering are not yet completely understood. However, trehalose-6-phosphate (T6P) signaling is critical for flowering transition regardless of day length [24]. T6P is produced from glucose-6-phosphate and uridine diphosphate (UDP)-glucose by the TREHALOSE-6-PHOSPHATE SYNTHASE 1 (TPS1) [182]. T6P cascade induces *FT* and its closest homolog *TSF* in a CO-independent manner [24]. In return, FT promotes the expression of the bidirectional sucrose transporter *SWEET 10* in the phloem companion Cells which is thought to be important for sugar export to the shoot apex [234]. Additionally, *TPS1* is highly expressed in the IM, and its overexpression reverts the late-flowering phenotype of *ft-10*, suggesting that T6P acts independently or downstream of FT regulation [24].

Under SD photoperiod, T6P positively regulates the expression of *SPL3/4/5* at the SAM in a partially independent manner of miR156 decreasing by aging [24]. However, there is also evidence that an increase in endogenous sugar levels represses miR156 [127,130,235]. This regulation could be partially mediated by the glucose sensor HEXOKINASE1 (HXK1) since the reduction of miR156 levels is compromised in the *hxk1* mutant in response to glucose [128].

The close relationship between SPL and MADS-box TFs, as well as their response to endogenous and environmental cues during plant’s phase transitions, strongly suggest that they are part of the same gene regulatory network (Figure 2). Moreover, it has been suggested that both groups of proteins act as pioneer transcription factors, binding and opening inaccessible chromatin by recruiting chromatin remodeling complexes [65,236].

## 5. Long Day Photoperiod and High Temperature Accelerate the Flowering Transition

Day length and high permissible temperatures are important signals to induce flowering, particularly for those plants that grow in latitudes where there are significant changes in photoperiod and temperatures throughout seasons. *A. thaliana* flowers under long-day photoperiod during spring and summer [237].

Detection of photoperiod relies primarily on CO protein, a B-box-type zinc finger TF with a CCT domain, that accumulates during the day in the vascular tissue [6,238]. *CO*’s mRNA is expressed in circadian cycles having its maximum accumulation levels around 16 h after the first light and dawn when *Arabidopsis* is grown under LD photoperiod [7]. This oscillating behavior is due to *CO*’s repression by CYCLING DOF FACTORs (CDFs) during the morning and the degradation of these factors in the late afternoon. The F-box E3-ubiquitin ligases FLAVIN BINDING, KELCH REPEAT, F-BOX1 (FKF1), and ZEITLUPE (ZTL) are photoreceptors, which in association with GI, target those CDFs for their degradation [103,239,240,241,242,243]. Furthermore, GI-FKF1 interaction is blue light-dependent, determining at least in part *CO* daytime expression [103]. GI itself is a circadian cycle protein [244,245] that induces *CO* expression in association with TEOSINTE BRANCHED 1/CYCLOIDEA/PROLIFERATING CELL NUCLEAR ANTIGEN FACTOR 4 (TCP4) [246]. Additionally, FLOWERING BHLH 1 to 4 (FBH1-4) TFs also upregulate *CO* expression [247].

At the posttranslational level, COP1 and SUPPRESSOR OF PHYA-105S 1 (COP1/SPA1) complex ubiquitinates CO protein to be degraded by the proteasome at night [248,249,250,251]. During the diurnal phase of LD-photoperiod, cry1, cry2, and phyA, are activated by blue and far-red light, respectively, inhibiting COP1/SPA1 activity. This action allows CO accumulation in the afternoon [248,252,253,254]. Consistent with this, the Cabo Verde islands (Cvi-0) accession plants carrying a gain-of-function allele of *CRY2*, are early flowering at 23 °C [255,256]. On the contrary, phyB and HIGH EXPRESSION OF OSMOTICALLY RESPONSIVE GENES 1 (HOS1) ubiquitinates CO in response to red light in the morning [253,257,258]. However, phyB inhibition can be counteracted by PHYTOCHROME-DEPENDENT LATE-FLOWERING (PHL) contributing to CO accumulation [259].

Interestingly, complex interconnections between GI, ZTL, and FKF1 proteins, shape CO protein accumulation during the day. GI-ZTL downregulates CO protein levels, while GI-FKF1 stabilizes them. Moreover, GI prevents ZTL-FKF1 union, favoring GI-FKF1 complex formation. These complex regulations lead to high CO protein levels in the late afternoon in LDs but not in SD [260,261]. Furthermore, FKF1 also ubiquitinates DELLA proteins freeing CO to induce *FT* and *SOC1* [206,262]. Additionally, FKF1 stabilizes CO activity, while TOE proteins might interfere with the FKF-CO association. Thus, CO accumulation is limited to LD afternoon, partly because TOEs levels decrease at this time [263].

**Figure 3:**
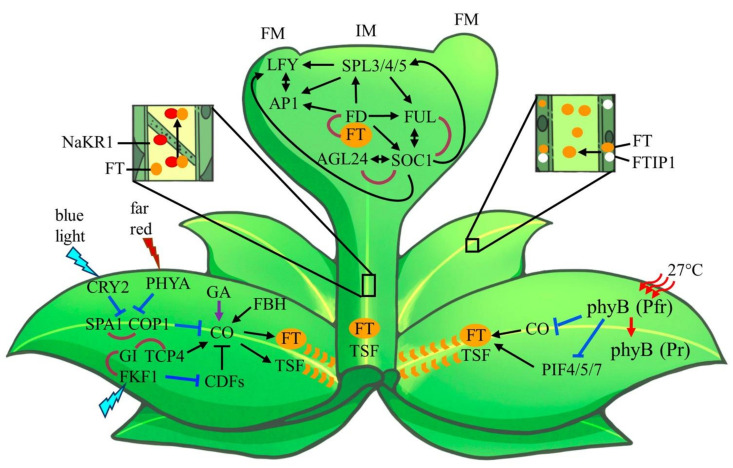
Flowering induction in response to LD photoperiod and high temperature. The transcriptional (black lines) and posttranslational regulation (blue lines) of CO mediates *FT* expression. phyA and cry2 promote CO protein’s stability, while phyB in its active state (Pfr) inhibits it. Thermal reversion of the latter favors the accumulation of CO and PIFs to induce *FT*. The FT protein travels through the phloem with the help of FTIP1 and NaKR. Once FT reaches the apical meristem, it associates with FD to induce some MADS-box genes, (including *AP1*) and SPLs that upregulate *LFY* and *AP1*. Magenta curve lines represent protein interactions.

CO induces *FT* expression by binding to two CO-responsive elements (CORE) in its promoter [26,264]. *FT* is translated in the companion cells of vascular tissue, and the protein constitutes a signal that travels from rosette leaves to the SAM to induce flowering [265,266]. Therefore, it acts as systemic florigen [267]. FT associates with FT-INTERACTING PROTEIN1 (FTIP1) to pass through plasmodesmata from the phloem companion cells to the sieve tubes [268]; next, FT induces and interacts with SODIUM POTASSIUM ROOT DEFECTIVE 1 (NaKR1), which is essential to reach long distances through the vascular system [269] (Figure 3).

FT [270,271] is one of the members of the phosphatidylethanolamine-binding protein (PEBP) family, that together with two other homologs, TSF and MOTHER OF FT (MFT), have redundant functions as flowering inducers [272,273]. Once they reach the SAM, FT and TSF interact with the bZIP transcription factor FD via the 14-3-3 growth response factors [274,275]. Although FD can bind to some genes even in the absence of FT or TSF, these enhance FD’s ability to upregulate *SOC1, FUL,* and *AP1* [275,276,277]. In this sense, FT functions as a transcriptional cofactor. Moreover, the FT-FD complex regulates the expression of *SPL3/4/5* in the IM [194,218], and they in turn, bind directly or in association with FD to *FUL, LFY*, and *AP1* regulatory regions to upregulate them [190,194] (Figure 3).

The FT-FD complex is transiently formed and disappears rapidly, at least in part, by limiting *FD* expression by AP1 in the MF [278]. Moreover, FT protein levels decrease probably by a proteolytic process [279].

CO induces *SOC1* in an FT-FD dependent and independent manner [26,280]. Furthermore, *FT* and *SOC1* are positively regulated by age, T6P, and GA signals [24,115,206,281] (Figure 2), and *SOC1* is indirectly induced by vernalization [28]. SOC1 and AGL24 mutually induce each other, and their dimer enters the nucleus, where it activates *LFY* expression [229,280]. Nonetheless, the triple mutant *ft soc1 lfy* still blooms under LDs, indicating there are other genes implicated [29]. Indeed, the MADS-domain protein AGAMOUS-LIKE 17 (AGL17) induces *AP1* in an FT independent mechanism [282].

LFY induces *AP1* and *CAL,* and in turn, AP1 upregulates *LFY* [283,284,285,286]. These feed-forward regulations reinsure FM identity.

In nature day length and temperature are two factors that usually go in the same direction. When winter ends and days become longer, the temperature also rises. Therefore, it is not surprising that warmer temperatures act as an inductive flowering signal in *Arabidopsis* plants. However, given climate change, an important question is whether a moderate rise in temperature could affect flowering time even when there is no variance in seasonal photoperiods [18]. Many *A. thaliana* accessions, including Columbia (Col-0) and Landsberg *erecta* (*Ler*) flower earlier when grown at 25–27 °C compared to 23 °C in SD photoperiod [20]. Moreover, this is not dependent on CO, since mutants in this gene still respond to thermal induction. However, the *ft-10* mutant is insensitive to warm temperature, showing that FT mediates flowering thermal response independently of LD photoperiod [20].

PHYTOCHROME INTERACTING FACTOR 4 (PIF 4) and its orthologs PIF5 and PIF7 are responsible for inducing *FT* and *TSF* expression in response to high temperature [287,288,289]. Interestingly, at 27 °C, the H2A.Z-nucleosomes levels decreased at the *FT* locus, relaxing the chromatin and favoring the union of PIF4 to *FT* DNA [287].

Red-light induces the phyB active Pfr state which promotes CO and PIF4 degradation, while a phenomenon called thermal reversion promotes fast change from the Pfr state to the Pr inactive state when temperature increases. This event allows both CO and PIF4 to induce *FT* [149,253,290,291] (Figure 3). Furthermore, flowering induction by high temperature requires GA in SD [20]. Since DELLAs repress PIF4 activity, high levels of GA induced by warmer temperatures could free PIF TFs to upregulate *FT* [287].

Noteworthy, FLM is required for thermal induction, in contrast to FLC that partially suppresses it. Thus, *Arabidopsis* accessions variability to flowering in response to thermal induction could be explained (at least in part) by genetic variation in those alleles [20]. It has been proposed that decreasing SVP and FLC levels at the meristem during flowering transition augments FT sensitivity to high temperature [20,292]. Moreover, low FLC levels lead to shortening the circadian period, which probably impacts flowering transition, especially on genes regulating photoperiodic responses [293].

The evidence suggests that LD photoperiod and warm temperature signaling can be separated, but more research is required to establish the latter’s influence when plants grow under LD photoperiod.

## 6. Concluding Remarks and Perspectives

A considerable effort has been made during decades to understand how plants regulate flowering transition in response to different seasons and changing climate conditions. The characterization of mutants that show early or late flowering phenotypes under specific growing conditions and their genetic relationships led to the genetic pathways’ classification. Although this approach is experimentally essential, the information summarized in this review highlights that the complex genetic network that underlies the transition to flowering transcends the genetic pathways.

The actual hierarchical flowering model proposed that different inputs converge into the integrators that transduce these signals to the FMI genes [1]. Alternatively, endogenous signals could transversally dictate whether the plants remain in the vegetative phase or initiate the reproductive state. In this view, miR156, the DELLA proteins, and possibly low concentration of certain carbohydrates maintain the vegetative state, while miR172, GA and T6P, allow the reproductive phase change. Inductive signals like LD-photoperiod and temperature accelerate the flowering transition process in plants such as *Arabidopsis*, in part, by upregulating *FT* in the leaves and a group of the SPLs and MADS-box genes in the apical meristem. This ensures that flowering happens when the external conditions are optimal for those species. Although the relevance of FT and SOC1 as flowering inducers is indisputable, there is current evidence that they are not the only ones that regulate *LFY* and *AP1* (Table 1), supporting the idea that not all the inputs converge into those two integrators.

Different regulation levels are relevant to establish the network’s developmental phase changes, including the epigenetic, transcriptional, posttranscriptional, and posttranslational regulation. At present, there is detailed information about some processes, while it is very little on others. With the evidence summarized here, it can be said that epigenetic regulation is important to maintain the vegetative phase by repressing the flowering inducers and, it is essential in flowering promotion by repressing *FLC* and other flowering repressors [38]. On the other hand, protein stabilization and turn-over rates are vital in accelerating flowering in response to LD photoperiod and high temperature [249]. Likewise, there is growing evidence showing that posttranscriptional regulation such as RNA processing and decay, non-coding RNAs and microRNAs, fine-tune flowering responses to specific conditions [294,295,296]. Notably, alternative splicing is a common regulatory mechanism that allows the plant to prevent or induce flowering depending on light and temperature rapidly [139,297,298,299,300,301]. Research into the mechanisms governing alternative splicing provides an exciting field to unravel regulatory mechanisms of plants’ environmental adaptation [57,291].

Despite our knowledge in the regulation of flowering, there are processes left to be uncovered. For example, it is still to know the signal transduction triggered by T6P, the effect of vernalization in a broad context, the possible signaling of photoreceptors in the apical meristem, and their activity as thermoreceptors. It is also necessary to continue studying how MADS-domain proteins associate in complexes whose combination affects their function [236,306,307].

Furthermore, there is much to learn from studies on natural variation, which have shown that *FLC, FLM*, and some circadian-cycle genes are fundamental for the adaptation of flowering time to different environments [54,308,309,310]. More comparative studies between *Arabidopsis* and other species will also expand our knowledge on common and divergent mechanisms on flowering regulation [311,312].

Finally, it would be important to continue implementing theoretical models to integrate the flowering information to infer regulations that cannot be easily detected. In this respect, different models have been developed [313,314,315]. However, it would be necessary to include more genes of the flowering network and simulate different growing conditions.

## Figures and Tables

**Table 1:** Transcriptional factors directly involved in *LFY* and *AP1* regulation.

Target	TFs	Regulation Type	Reference
***LFY***	AGL24	Positive	[302]
	AP1	Positive	[285]
	FD	Positive/Negative	[194]
	FUL	Positive	[226]
	LFY	Positive	[303]
	MYB33	Positive	[230]
	PNY	Positive	[236]
	SOC1	Positive	[229]
	SPL10	Positive	[193]
	SPL3	Positive	[190]
	SVP	Positive	[302]
	WRKY71	Positive	[222]
***AP1***	AGL24	Positive	[302]
	AP1	Positive	[304]
	AP2	Negative	[97]
	CAL	Positive	[304]
	FD	Positive/Negative	[194]
	LFY	Positive	[284]
	PNY	Positive	[236]
	RGA	Positive	[219]
	SMZ	Negative	[47]
	SPL3	Positive	[190]
	SPL9	Positive	[305]
	SVP	Positive	[302]

## References

[1] Blázquez M.A., Ferrándiz C., Madueño F., Parcy F. (2006). How Floral Meristems Are Built. Plant Mol. Biol..

[2] Benlloch R., Berbel A., Serrano-Mislata A., Madueño F. (2007). Floral Initiation and Inflorescence Architecture: A Comparative View. Ann. Bot..

[3] Coen E.S., Meyerowitz E.M. (1991). The War of the Whorls: Genetic Interactions Controlling Flower Development. Nature.

[4] Ausín I., Alonso-Blanco C., Martínez-Zapater J.M. (2005). Environmental Regulation of Flowering. Int. J. Develop. Biol..

[5] Koornneef M., Hanhart C.J., van der Veen J.H. (1991). A Genetic and Physiological Analysis of Late Flowering Mutants in Arabidopsis Thaliana. MGG Mol. Gen. Genet..

[6] Putterill J., Robson F., Lee K., Simon R., Coupland G. (1995). The CONSTANS Gene of Arabidopsis Promotes Flowering and Encodes a Protein Showing Similarities to Zinc Finger Transcription Factors. Cell.

[7] Suárez-López P.K., Wheatley F., Robson H., Onouchi F., Valverde G. (2001). Coupland. CONSTANS Mediates between the Circadian Clock and the Control of Flowering in Arabidopsis. Nature.

[8] Imaizumi T., Kay S.A. (2006). Photoperiodic Control of Flowering: Not Only by Coincidence. Trends Plant Sci..

[9] Blázquez M.A., Green R., Nilsson O., Sussman M.R., Weigel D. (1998). Gibberellins Promote Flowering of Arabidopsis by Activating the LEAFY Promoter. Plant Cell.

[10] Blazquez M.A., Weigel D. (2000). Integration of Floral Inductive Signals in Arabidopsis. Nature.

[11] Porri A., Torti S., Romera-Branchat M., Coupland G. (2012). Spatially Distinct Regulatory Roles for Gibberellins in the Promotion of Flowering of Arabidopsis under Long Photoperiods. Development.

[12] Michaels S.D., Amasino R.M. (1999). The Gibberellic Acid Biosynthesis Mutant Ga1-3 of Arabidopsis Thaliana Is Responsive to Vernalization. Develop. Genet..

[13] Sheldon C.C., Rouse D.T., Finnegan E.J., Peacock W.J., Dennis E.S. (2000). The Molecular Basis of Vernalization: The Central Role of FLOWERING LOCUS C (FLC). Proc. Natl. Acad. Sci. USA.

[14] He Y., Michaels S.D., Amasino R.M. (2003). Regulation of Flowering Time by Histone Acetylation in Arabidopsis. Science.

[15] Simpson G.G. (2004). The Autonomous Pathway: Epigenetic and Post-Transcriptional Gene Regulation in the Control of Arabidopsis Flowering Time. Curr. Opin. Plant Biol..

[16] Koornneef M., Alonso-Blanco C., Blankestijn-De Vries H., Hanhart C.J., Peeters A.J.M. (1998). Genetic Interactions among Late-Flowering Mutants of Arabidopsis. Genetics.

[17] Pajoro A., Biewers S., Dougali E., Valentim F.L., Mendes M.A., Porri A., Coupland G., Van De Peer Y., Van Dijk A.D.J., Colombo L. (2014). The (r)Evolution of Gene Regulatory Networks Controlling Arabidopsis Plant Reproduction: A Two-Decade History. J. Exp. Bot..

[18] Blázquez M.A., Ahn J.H., Weigel D. (2003). A Thermosensory Pathway Controlling Flowering Time in Arabidopsis Thaliana. Nat. Genet..

[19] Halliday K.J., Salter M.G., Thingnaes E., Whitelam G.C. (2003). Phytochrome Control of Flowering Is Temperature Sensitive and Correlates with Expression of the Floral Integrator FT. Plant J..

[20] Balasubramanian S., Sureshkumar S., Lempe J., Weigel D. (2006). Potent Induction of Arabidopsis Thaliana Flowering by Elevated Growth Temperature. PLoS Genet..

[21] Lee J.H., Yoo S.J., Park S.H., Hwang I., Lee J.S., Ahn J.H. (2007). Role of SVP in the Control of Flowering Time by Ambient Temperature in Arabidopsis. Genes Develop..

[22] Wu G., Poethig R.S. (2006). Temporal Regulation of Shoot Development in Arabidopsis Thaliana by MiRr156 and Its Target SPL3. Development.

[23] Schwarz S., Grande A.V., Bujdoso N., Saedler H., Huijser P. (2008). The MicroRNA Regulated SBP-Box Genes SPL9 and SPL15 Control Shoot Maturation in Arabidopsis. Plant Mol. Biol..

[24] Wahl V., Ponnu J., Schlereth A., Arrivault S., Langenecker T., Franke A., Feil R., Lunn J.E., Stitt M., Schmid M. (2013). Regulation of Flowering by Trehalose-6-Phosphate Signaling in Arabidopsis Thaliana. Science.

[25] Lee H., Suh S.S., Park E., Cho E., Ahn J.H., Kim S.G., Lee J.S., Kwon Y.M., Lee I. (2000). The AGAMOUS-LIKE 20 MADS Domain Protein Integrates Floral Inductive Pathways in Arabidopsis. Genes Develop..

[26] Samach A., Onouchi H., Gold S.E., Ditta G.S., Schwarz-Sommer Z., Yanofsky M.F., Coupland G. (2000). Distinct Roles of Constans Target Genes in Reproductive Development of Arabidopsis. Science.

[27] Hepworth S.R., Valverde F., Ravenscroft D., Mouradov A., Coupland G. (2002). Antagonistic Regulation of Flowering-Time Gene SOC1 by CONSTANS and FLC via Separate Promoter Motifs. EMBO J..

[28] Moon J., Suh S.S., Lee H., Choi K.R., Hong C.B., Paek N.C., Kim S.G., Lee I. (2003). The SOC1 MADS-Box Gene Integrates Vernalization and Gibberellin Signals for Flowering in Arabidopsis. Plant J..

[29] Moon J., Lee H., Kim M., Lee I. (2005). Analysis of Flowering Pathway Integrators in Arabidopsis. Plant Cell Physiol..

[30] Michaels S.D., Himelblau E., Sang Y.K., Schomburg F.M., Amasino R.M. (2005). Integration of Flowering Signals in Winter-Annual Arabidopsis. Plant Physiol..

[31] Hempel F.D., Weigel D., Alejandra Mandel M., Ditta G., Zambryski P.C., Feldman L.J., Yanofsky M.F. (1997). Floral Determination and Expression of Floral Regulatory Genes in Arabidopsis. Development.

[32] Bowman J.L., Alvarez J., Weigel D., Meyerowitz E.M., Smyth D.R. (1993). Control of Flower Development in Arabidopsis Thaliana by APETALA 1 and Interacting Genes. Development.

[33] Gregis V., Sessa A., Colombo L., Kater M.M. (2008). AGAMOUS-LIKE24 and SHORT VEGETATIVE PHASE Determine Floral Meristem Identity in Arabidopsis. Plant J..

[34] Liu C., Xi W., Shen L., Tan C., Yu H. (2009). Regulation of Floral Patterning by Flowering Time Genes. Develop. Cell.

[35] Blümel M., Dally N., Jung C. (2015). Flowering Time Regulation in Crops-What Did We Learn from Arabidopsis?. Curr. Opin. Biotechnol..

[36] Bouché F., Lobet G., Tocquin P., Périlleux C. (2016). FLOR-ID: An Interactive Database of Flowering-Time Gene Networks in Arabidopsis Thaliana. Nucleic Acids Res..

[37] Srikanth A., Schmid M. (2011). Regulation of Flowering Time: All Roads Lead to Rome. Cell. Mol. Life Sci..

[38] Berry S., Dean C. (2015). Environmental Perception and Epigenetic Memory: Mechanistic Insight through FLC. Plant J..

[39] Hyun Y., Richter R., Coupland G. (2017). Competence to Flower: Age-Controlled Sensitivity to Environmental Cues. Plant Physiol..

[40] Perrella G., Vellutini E., Zioutopoulou A., Patitaki E., Headland L.R., Kaiserli E. (2020). Let It Bloom: Cross-Talk between Light and Flowering Signaling in Arabidopsis. Physiol. Plant..

[41] Koornneef M., Blankestijn-de Vries H., Hanhart C., Soppe W., Peeters T. (1994). The Phenotype of Some Late-flowering Mutants Is Enhanced by a Locus on Chromosome 5 That Is Not Effective in the Landsberg Erecta Wild-type. Plant J..

[42] Sheldon C.C., Burn J.E., Perez P.P., Metzger J., Edwards J.A., Peacock W.J., Dennis E.S. (1999). The FLF MADS Box Gene: A Repressor of Flowering in Arabidopsis Regulated by Vernalization and Methylation. Plant Cell.

[43] Hartmann U., Höhmann S., Nettesheim K., Wisman E., Saedler H., Huijser P. (2000). Molecular Cloning of SVP: A Negative Regulator of the Floral Transition in Arabidopsis. Plant J..

[44] Ratcliffe O.J., Nadzan G.C., Reuber T.L., Riechmann J.L. (2001). Regulation of Flowering in Arabidopsis by an FLC Homologue. Plant Physiol..

[45] Scortecci K.C., Michaels S.D., Amasino R.M. (2001). Identification of a MADS-Box Gene FLOWERING LOCUS M, That Represses Flowering. Plant J..

[46] Adamczyk B.J., Lehti-Shiu M.D., Fernandez D.E. (2007). The MADS Domain Factors AGL15 and AGL18 Act Redundantly as Repressors of the Floral Transition in Arabidopsis. Plant J..

[47] Mathieu J., Yant L.J., Mürdter F., Küttner F., Schmid M. (2009). Repression of Flowering by the MiR172 Target SMZ. PLoS Biol..

[48] Deng W., Ying H., Helliwell C.A., Taylor J.M., Peacock W.J., Dennis E.S. (2011). FLOWERING LOCUS C (FLC) Regulates Development Pathways throughout the Life Cycle of Arabidopsis. Proc. Natl. Acad. Sci. USA.

[49] Willmann M.R., Poethig R.S. (2011). The Effect of the Floral Repressor FLC on the Timing and Progression of Vegetative Phase Change in Arabidopsis. Development.

[50] Chen M., Penfield S. (2018). Feedback Regulation of COOLAIR Expression Controls Seed Dormancy and Flowering Time. Science.

[51] Helliwell C.A., Wood C.C., Robertson M., James Peacock W., Dennis E.S. (2006). The Arabidopsis FLC Protein Interacts Directly in Vivo with SOC1 and FT Chromatin and Is Part of a High-Molecular-Weight Protein Complex. Plant J..

[52] Searle I., He Y., Turck F., Vincent C., Fornara F., Kröber S., Amasino R.A., Coupland G. (2006). The Transcription Factor FLC Confers a Flowering Response to Vernalization by Repressing Meristem Competence and Systemic Signaling in Arabidopsis. Genes Develop..

[53] Luo X., Chen T., Zeng X., He D., He Y. (2019). Feedback Regulation of FLC by FLOWERING LOCUS T (FT) and FD through a 5′ FLC Promoter Region in Arabidopsis. Mol. Plant.

[54] Fujiwara S., Oda A., Yoshida R., Niinuma K., Miyata K., Tomozoe Y., Tajima T., Nakagawa M., Hayashi K., Coupland G. (2008). Circadian Clock Proteins LHY and CCA1 Regulate SVP Protein Accumulation to Control Flowering in Arabidopsis. Plant Cell.

[55] Li D., Liu C., Shen L., Wu Y., Chen H., Robertson M., Helliwell C.A., Ito T., Meyerowitz E., Yu H. (2008). A Repressor Complex Governs the Integration of Flowering Signals in Arabidopsis. Develop. Cell.

[56] Mateos J.L., Madrigal P., Tsuda K., Rawat V., Richter R., Romera-Branchat M., Fornara F., Schneeberger K., Krajewski P., Coupland G. (2015). Combinatorial Activities of Short Vegetative Phase And Flowering Locus C Define Distinct Modes of Flowering Regulation in Arabidopsis. Genome Biol..

[57] Theißen G., Rümpler F., Gramzow L. (2018). Array of MADS-Box Genes: Facilitator for Rapid Adaptation?. Trends Plant Sci..

[58] Jang S., Torti S., Coupland G. (2009). Genetic and Spatial Interactions between FT, TSF and SVP during the Early Stages of Floral Induction in Arabidopsis. Plant J..

[59] Gu X., Le C., Wang Y., Li Z., Jiang D., Wang Y., He Y. (2013). Arabidopsis FLC Clade Members Form Flowering-Repressor Complexes Coordinating Responses to Endogenous and Environmental Cues. Nat. Commun..

[60] Lee J.H., Ryu H.-S., Chung K.S., Pose D., Kim S., Schmid M., Ahn J.H. (2013). Regulation of Temperature-Responsive Flowering by MADS-Box Transcription Factor Repressors. Science.

[61] Posé D., Verhage L., Ott F., Yant L., Mathieu J., Angenent G.C., Immink R.G.H., Schmid M. (2013). Temperature-Dependent Regulation of Flowering by Antagonistic FLM Variants. Nature.

[62] Tao Z., Shen L., Liu C., Liu L., Yan Y., Yu H. (2012). Genome-Wide Identification of SOC1 and SVP Targets during the Floral Transition in Arabidopsis. Plant J..

[63] Gregis V., Andrés F., Sessa A., Guerra R.F., Simonini S., Mateos J.L., Torti S., Zambelli F., Prazzoli G.M., Bjerkan K.N. (2013). Identification of Pathways Directly Regulated by short vegetative phase during Vegetative and Reproductive Development in Arabidopsis. Genome Biol..

[64] Porri A., Torti S., Mateos J., Romera-Branchat M., García-Martínez J.L., Fornara F., Gregis V., Kater M.M., Coupland G. (2014). SHORT VEGETATIVE PHASE Reduces Gibberellin Biosynthesis at the Arabidopsis Shoot Apex to Regulate the Floral Transition. Proc. Natl. Acad. Sci. USA.

[65] Richter R., Kinoshita A., Vincent C., Martinez-Gallegos R., Gao H., van Driel A.D., Hyun Y., Mateos J.L., Coupland G. (2019). Floral Regulators FLC and SOC1 Directly Regulate Expression of the B3-Type Transcription Factor TARGET of FLC and SVP 1 at the Arabidopsis Shoot Apex via Antagonistic Chromatin Modifications. PLoS Genet..

[66] Li H., Ye K., Shi Y., Cheng J., Zhang X., Yang S. (2017). BZR1 Positively Regulates Freezing Tolerance via CBF-Dependent and CBF-Independent Pathways in Arabidopsis. Mol. Plant.

[67] Yu C.W., Liu X., Luo M., Chen C., Lin X., Tian G., Lu Q., Cui Y., Wu K. (2011). HISTONE DEACETYLASE6 Interacts with FLOWERING LOCUS D and Regulates Flowering in Arabidopsis. Plant Physiol..

[68] Luo M., Tai R., Yu C.W., Yang S., Chen C.Y., Lin W.D., Schmidt W., Wu K. (2015). Regulation of Flowering Time by the Histone Deacetylase HDA5 in Arabidopsis. Plant J..

[69] Cheng J.Z., Zhou Y.P., Lv T.X., Xie C.P., Tian C.E. (2017). Research Progress on the Autonomous Flowering Time Pathway in Arabidopsis. Physiol. Mol. Biol. Plants.

[70] Ausín I., Alonso-Blanco C., Jarillo J.A., Ruiz-García L., Martínez-Zapater J.M. (2004). Regulation of Flowering Time by FVE, a Retinoblastoma-Associated Protein. Nat. Genet..

[71] Noh B., Lee S.H., Kim H.J., Yi G., Shin E.A., Lee M., Jung K.J., Doyle M.R., Amasino R.M., Noh Y.S. (2004). Divergent Roles of a Pair of Homologous Jumonji/Zinc-Finger-Class Transcription Factor Proteins in the Regulation of Arabidopsis Flowering Time. Plant Cell.

[72] Domagalska M.A., Schomburg F.M., Amasino R.M., Vierstra R.D., Nagy F., Davis S.J. (2007). Attenuation of Brassinosteroid Signaling Enhances FLC Expression and Delays Flowering. Development.

[73] Niu L., Lu F., Pei Y., Liu C., Cao X. (2007). Regulation of Flowering Time by the Protein Arginine Methyltransferase AtPRMT10. EMBO Rep..

[74] Hornyik C., Duc C., Rataj K., Terzi L.C., Simpson G.G. (2010). Alternative Polyadenylation of Antisense RNAs and Flowering Time Control. Biochem. Soc. Trans..

[75] Zhong X., Du J., Hale C.J., Gallego-Bartolome J., Feng S., Vashisht A.A., Chory J., Wohlschlegel J.A., Patel D.J., Jacobsen S.E. (2014). Molecular Mechanism of Action of Plant DRM de Novo DNA Methyltransferases. Cell.

[76] Lee I., Michaels S.D., Masshardt A.S., Amasino R.M. (1994). The Late-flowering Phenotype of FRIGIDA and Mutations in LUMINIDEPENDENS Is Suppressed in the Landsberg Erecta Strain of Arabidopsis. Plant J..

[77] Macknight R., Bancroft I., Page T., Lister C., Schmidt R., Love K., Westphal L., Murphy G., Sherson S., Cobbett C. (1997). FCA, a Gene Controlling Flowering Time in Arabidopsis, Encodes a Protein Containing RNA-Binding Domains. Cell.

[78] Schomburg F.M., Patton D.A., Meinke D.W., Amasino R.M. (2001). FPA, a Gene Involved in Floral Induction in Arabidopsis, Encodes a Protein Containing RNA-Recognition Motifs. Plant Cell.

[79] Lim M.H., Kim J., Kim Y.S., Chung K.S., Seo Y.H., Lee I., Kim J., Hong C.B., Kim H.J., Park C.M. (2004). A New Arabidopsis Gene FLK, Encodes an RNA Binding Protein with K Homology Motifs and Regulates Flowering Time via Flowering Locus C. Plant Cell.

[80] Simpson G.G., Dijkwel P.P., Quesada V., Henderson I., Dean C. (2003). FY Is an RNA 3′ End-Processing Factor That Interacts with FCA to Control the Arabidopsis Floral Transition. Cell.

[81] Marquardt S., Boss P.K., Hadfield J., Dean C. (2006). Additional Targets of the Arabidopsis Autonomous Pathway Members, FCA and FY. J. Exp. Bot..

[82] Xing D., Zhao H., Xu R., Li Q.Q. (2008). Arabidopsis PCFS4, a Homologue of Yeast Polyadenylation Factor Pcf11p, Regulates FCA Alternative Processing and Promotes Flowering Time. Plant J..

[83] Wu Z., Fang X., Zhu D., Dean C. (2020). Autonomous Pathway: Flowering Locus c Repression through an Antisense-Mediated Chromatin-Silencing Mechanism. Plant Physiol..

[84] José Ripoll J., Ferrándiz C., Martínez-Laborda A., Vera A. (2006). PEPPER, a Novel K-Homology Domain Gene Regulates Vegetative and Gynoecium Development in Arabidopsis. Develop. Biol..

[85] Doyle M.R., Bizzell C.M., Keller M.R., Michaels S.D., Song J., Non Y.S., Amasino R.M. (2005). HUA2 Is Required for the Expression of Floral Repressors in Arabidopsis Thaliana. Plant J..

[86] Ortuño-Miquel S., Rodríguez-Cazorla E., Zavala-Gonzalez E.A., Martínez-Laborda A., Vera A. (2019). Arabidopsis HUA ENHANCER 4 Delays Flowering by Upregulating the MADS-Box Repressor Genes FLC and MAF4. Sci. Rep..

[87] Marquardt S., Raitskin O., Wu Z., Liu F., Sun Q., Dean C. (2014). Functional Consequences of Splicing of the Antisense Transcript COOLAIR on FLC Transcription. Mol. Cell.

[88] Liu F., Quesada V., Crevillén P., Bäurle I., Swiezewski S., Dean C. (2007). The Arabidopsis RNA-Binding Protein FCA Requires a Lysine-Specific Demethylase 1 Homolog to Downregulate FLC. Mol. Cell.

[89] Tian Y., Zheng H., Zhang F., Wang S., Ji X., Xu C., He Y., Ding Y. (2019). PRC2 Recruitment and H3K27me3 Deposition at FLC Require FCA Binding of COOLAIR. Sci. Adv..

[90] Mulekar J.J., Huq E. (2012). Does CK2 Affect Flowering Time by Modulating the Autonomous Pathway in Arabidopsis?. Plant Signal. Behav..

[91] Heidari B., Nemie-Feyissa D., Kangasjärvi S., Lillo C. (2013). Antagonistic Regulation of Flowering Time through Distinct Regulatory Subunits of Protein Phosphatase 2A. PLoS ONE.

[92] Robertson M., Helliwell C.A., Dennis E.S. (2008). Post-Translational Modifications of the Endogenous and Transgenic FLC Protein in Arabidopsis Thaliana. Plant Cell Physiol..

[93] Kwak J.S., Son G.H., Kim S.I., Song J.T., Seo H.S. (2016). Arabidopsis HIGH PLOIDY2 Sumoylates and Stabilizes Flowering Locus C through Its E3 Ligase Activity. Front. Plant Sci..

[94] Sureshkumar S., Dent C., Seleznev A., Tasset C., Balasubramanian S. (2016). Nonsense-Mediated MRNA Decay Modulates FLM-Dependent Thermosensory Flowering Response in Arabidopsis. Nat. Plants.

[95] Shen L., Kang Y.G.G., Liu L., Yu H. (2011). The J-Domain Protein J3 Mediates the Integration of Flowering Signals in Arabidopsis. Plant Cell.

[96] Cho H.J., Kim J.J., Lee J.H., Kim W., Jung J.H., Park C.M., Ahn J.H. (2012). SHORT VEGETATIVE PHASE (SVP) Protein Negatively Regulates MiR172 Transcription via Direct Binding to the Pri-MiR172a Promoter in Arabidopsis. FEBS Lett..

[97] Yant L., Mathieu J., Dinh T.T., Ott F., Lanz C., Wollmann H., Chen X., Schmid M. (2010). Orchestration of the Floral Transition and Floral Development in Arabidopsis by the Bifunctional Transcription Factor APETALA2. Plant Cell.

[98] Jung J.H., Seo Y.H., Pil J.S., Reyes J.L., Yun J., Chua N.H., Park C.M. (2007). The GIGANTEA-Regulated MicroRNA172 Mediates Photoperiodic Flowering Independent of CONSTANS in Arabidopsis. Plant Cell.

[99] Castillejo C., Pelaz S. (2008). The Balance between CONSTANS and TEMPRANILLO Activities Determines FT Expression to Trigger Flowering. Curr. Biol..

[100] Aguilar-Jaramillo A.E., Marín-González E., Matías-Hernández L., Osnato M., Pelaz S., Suárez-López P. (2019). TEMPRANILLO Is a Direct Repressor of the MicroRNA MiR172. Plant J..

[101] Osnato M., Castillejo C., Matías-Hernández L., Pelaz S. (2012). TEMPRANILLO Genes Link Photoperiod and Gibberellin Pathways to Control Flowering in Arabidopsis. Nat. Commun..

[102] Sawa M., Kay S.A. (2011). GIGANTEA Directly Activates Flowering Locus T in Arabidopsis Thaliana. Proc. Natl. Acad. Sci. USA.

[103] Sawa M., Nusinow D.A., Kay S.A., Imaizumi T. (2007). FKF1 and GIGANTEA Complex Formation Is Required for Day-Length Measurement in Arabidopsis. Science.

[104] Ritter A., Iñigo S., Fernández-Calvo P., Heyndrickx K.S., Dhondt S., Shi H., De Milde L., Vanden Bossche R., De Clercq R., Eeckhout D. (2017). The Transcriptional Repressor Complex FRS7-FRS12 Regulates Flowering Time and Growth in Arabidopsis. Nat. Commun..

[105] Fernandez D.E., Wang C.T., Zheng Y., Adamczyk B.J., Singhal R., Hall P.K., Perry S.E. (2014). The MADS-Domain Factors AGAMOUS-LIKE15 and AGAMOUS-LIKE18, along with SHORT VEGETATIVE PHASE and AGAMOUS-LIKE24, Are Necessary to Block Floral Gene Expression during the Vegetative Phase. Plant Physiol..

[106] Serivichyaswat P., Ryu H.S., Kim W., Kim S., Chung K.S., Kim J.J., Ahn J.H. (2015). Expression of the Floral Repressor MiRNA156 Is Positively Regulated by the AGAMOUS-like Proteins AGL15 and AGL18. Mol. Cells.

[107] Zheng Y., Ren N., Wang H., Stromberg A.J., Perry S.E. (2009). Global Identification of Targets of the Arabidopsis MADS Domain Protein AGAMOUS-Like15. Plant Cell.

[108] Del Olmo I., López-González L., Martín-Trillo M.M., Martínez-Zapater J.M., Piñeiro M., Jarillo J.A. (2010). EARLY IN SHORT DAYS 7 (ESD7) Encodes the Catalytic Subunit of DNA Polymerase Epsilon and Is Required for Flowering Repression through a Mechanism Involving Epigenetic Gene Silencing. Plant J..

[109] Del Olmo I., Lopez J.A., Vazquez J., Raynaud C., Pineiro M., Jarillo J.A. (2016). Arabidopsis DNA Polymerase ϵ Recruits Components of Polycomb Repressor Complex to Mediate Epigenetic Gene Silencing. Nucleic Acids Res..

[110] Kotake T., Takada S., Nakahigashi K., Ohto M., Goto K. (2003). Arabidopsis Terminal Flower 2 Gene Encodes a Heterochromatin Protein 1 Homolog and Represses Both FLOWERING LOCUS T to Regulate Flowering Time and Several Floral Homeotic Genes. Plant Cell Physiol..

[111] Mozgova I., Hennig L. (2015). The Polycomb Group Protein Regulatory Network. Annu. Rev. Plant Biol..

[112] Mylne J.S., Barrett L., Tessadori F., Mesnage S., Johnson L., Bernatavichute Y.V., Jacobsen S.E., Fransz P., Dean C. (2006). LHP1, the Arabidopsis Homologue of HETEROCHROMATIN PROTEIN1, Is Required for Epigenetic Silencing of FLC. Proc. Natl. Acad. Sci. USA.

[113] Sung S., He Y., Eshoo T.W., Tamada Y., Johnson L., Nakahigashi K., Goto K., Jacobsen S.E., Amasino R.M. (2006). Epigenetic Maintenance of the Vernalized State in Arabidopsis Thaliana Requires like heterochromatin protein 1. Nat. Genet..

[114] Turck F., Roudier F., Farrona S., Martin-Magniette M.L., Guillaume E., Buisine N., Gagnot S., Martienssen R.A., Coupland G., Colot V. (2007). Arabidopsis TFL2/LHP1 Specifically Associates with Genes Marked by Trimethylation of Histone H3 Lysine 27. PLoS Genet..

[115] Hou X., Zhou J., Liu C., Liu L., Shen L., Yu H. (2014). Nuclear Factor Y-Mediated H3K27me3 Demethylation of the SOC1 Locus Orchestrates Flowering Responses of Arabidopsis. Nat. Commun..

[116] Derkacheva M., Steinbach Y., Wildhaber T., Mozgová I., Mahrez W., Nanni P., Bischof S., Gruissem W., Hennig L. (2013). Arabidopsis MSI1 Connects LHP1 to PRC2 Complexes. EMBO J..

[117] Gómez-Mena C., Piñeiro M., Franco-Zorrilla J.M., Salinas J., Coupland G., Martínez-Zapater J.M. (2001). Early Bolting in Short Days: An Arabidopsis Mutation That Causes Early Flowering and Partially Suppresses the Floral Phenotype of Leafy. Plant Cell.

[118] López-González L., Mouriz A., Narro-Diego L., Bustos R., Martínez-Zapater J.M., Jarillo J.A., Piñeiro M. (2014). Chromatin-Dependent Repression of the Arabidopsis Floral Integrator Genes Involves Plant Specific PHD-Containing Proteins. Plant Cell.

[119] Yang Z., Qian S., Scheid R.N., Lu L., Chen X., Du X., Lv X., Boersma M.D., Scalf M., Smith L.M. (2019). EBS Is a Bivalent Histone Reader That Regulates Floral Phase Transition in Arabidopsis. Nat. Genet..

[120] Li Z., Fu X., Wang Y., Liu R., He Y. (2018). Polycomb-Mediated Gene Silencing by the BAH–EMF1 Complex in Plants. Nat. Genet..

[121] Huang F., Yuan W., Tian S., Zheng Q., He Y. (2019). SIN3 LIKE Genes Mediate Long-Day Induction of Flowering but Inhibit the Floral Transition in Short Days through Histone Deacetylation in Arabidopsis. Plant J..

[122] Sang Q., Pajoro A., Sun H., Song B., Yang X., Stolze S.C., Andrés F., Schneeberger K., Nakagami H., Coupland G. (2020). Mutagenesis of a Quintuple Mutant Impaired in Environmental Responses Reveals Roles for Chromatin Remodeling4 in the Arabidopsis Floral Transition. Plant Cell.

[123] Wu G., Park M.Y., Conway S.R., Wang J.W., Weigel D., Poethig R.S. (2009). The Sequential Action of MiR156 and MiR172 Regulates Developmental Timing in Arabidopsis. Cell.

[124] Fouracre J.P., Poethig R.S. (2019). Role for the Shoot Apical Meristem in the Specification of Juvenile Leaf Identity in Arabidopsis. Proc. Natl. Acad. Sci. USA.

[125] Xu M., Hu T., Smith M.R., Poethig R.S. (2016). Epigenetic Regulation of Vegetative Phase Change in Arabidopsis. Plant Cell.

[126] Xu Y., Guo C., Zhou B., Li C., Wang H., Zheng B., Ding H., Zhu Z., Peragine A., Cui Y. (2016). Regulation of Vegetative Phase Change by SWI2/SNF2 Chromatin Remodeling ATPase BRAHMA. Plant Physiol..

[127] Yu S., Li C., Zhou C.M., Zhang T.Q., Lian H., Sun Y., Wu J., Huang J., Wang G., Wang J.W. (2013). Sugar Is an Endogenous Cue for Juvenile-to-Adult Phase Transition in Plants. Elife.

[128] Yang L., Xu M., Koo Y., He J., Scott Poethig R. (2013). Sugar Promotes Vegetative Phase Change in Arabidopsis Thaliana by Repressing the Expression of MIR156A and MIR156C. Elife.

[129] Stewart Gillmor C., Silva-Ortega C.O., Willmann M.R., Buendía-Monreal M., Poethig R.S. (2014). The Arabidopsis Mediator CDK8 Module Genes CCT (MED12) and GCT (MED13) Are Global Regulators of Developmental Phase Transitions. Development.

[130] Buendía-Monreal M., Gillmor C.S. (2017). Convergent Repression of MiR156 by Sugar and the CDK8 Module of Arabidopsis Mediator. Develop. Biol..

[131] Jung J.H., Seo P.J., Ahn J.H., Park C.M. (2012). Arabidopsis RNA-Binding Protein FCA Regulates MicroRNA172 Processing in Thermosensory Flowering. J. Biol. Chem..

[132] Chen X. (2004). A MicroRNA as a Translational Repressor of APETALA2 in Arabidopsis Flower Development. Science.

[133] Hemming M.N., Trevaskis B. (2011). Make Hay When the Sun Shines: The Role of MADS-Box Genes in Temperature-Dependant Seasonal Flowering Responses. Plant Sci..

[134] Bouché F., Woods D.P., Amasino R.M. (2017). Winter Memory throughout the Plant Kingdom: Different Paths to Flowering. Plant Physiol..

[135] Lutz U., Nussbaumer T., Spannagl M., Diener J., Mayer K.F.X., Schwechheimer C. (2017). Natural Haplotypes of FLM Non-Coding Sequences Fine-Tune Flowering Time in Ambient Spring Temperatures in Arabidopsis. Elife.

[136] Bloomer R.H., Dean C. (2017). Fine-Tuning Timing: Natural Variation Informs the Mechanistic Basis of the Switch to Flowering in Arabidopsis Thaliana. J. Exp. Bot..

[137] Song Y.H., Ito S., Imaizumi T. (2013). Flowering Time Regulation: Photoperiod- and Temperature-Sensing in Leaves. Trends Plant Sci..

[138] Li L., Li X., Liu Y., Liu H. (2016). Flowering Responses to Light and Temperature. Sci. China Life Sci..

[139] Lee K.C., Chung K.S., Lee H.T., Park J.H., Lee J.H., Kim J.K. (2020). Role of Arabidopsis Splicing Factor SF1 in Temperature-Responsive Alternative Splicing of FLM Pre-MRNA. Front. Plant Sci..

[140] Capovilla G., Symeonidi E., Wu R., Schmid M. (2017). Contribution of Major FLM Isoforms to Temperature-Dependent Flowering in Arabidopsis Thaliana. J. Exp. Bot..

[141] Lutz U., Posé D., Pfeifer M., Gundlach H., Hagmann J., Wang C., Weigel D., Mayer K.F.X., Schmid M., Schwechheimer C. (2015). Modulation of Ambient Temperature-Dependent Flowering in Arabidopsis Thaliana by Natural Variation of Flowering Locus M. PLoS Genet..

[142] Ratcliffe O.J., Kumimoto R.W., Wong B.J., Riechmann J.L. (2003). Analysis of the Arabidopsis MADS AFFECTING FLOWERING Gene Family: MAF2 Prevents Vernalization by Short Periods of Cold. Plant Cell.

[143] Rosloski S.M., Singh A., Jali S.S., Balasubramanian S., Weigel D., Grbic V. (2013). Functional Analysis of Splice Variant Expression of MADS AFFECTING FLOWERING 2 of Arabidopsis Thaliana. Plant Mol. Biol..

[144] Airoldi C.A., McKay M., Davies B. (2015). MAF2 Is Regulated by Temperature-Dependent Splicing and Represses Flowering at Low Temperatures in Parallel with FLM. PLoS ONE.

[145] Macknight R., Duroux M., Laurie R., Dijkwel P., Simpson G., Dean C. (2002). Functional Significance of the Alternative Transcript Processing of the Arabidopsis Floral Promoter FCA. Plant Cell.

[146] Quesada V., Macknight R., Dean C., Simpson G.G. (2003). Autoregulation of FCA Pre-MRNA Processing Controls Arabidopsis Flowering Time. EMBO J..

[147] Lee H., Yoo S.J., Lee J.H., Kim W., Yoo S.K., Fitzgerald H., Carrington J.C., Ahn J.H. (2010). Genetic Framework for Flowering-Time Regulation by Ambient Temperature-Responsive MiRNAs in Arabidopsis. Nucleic Acids Res..

[148] Kim J.J., Lee J.H., Kim W., Jung H.S., Huijser P., Ahn J.H. (2012). The MicroRNA 156-SQUAMOSA Promoter Binding Protein-Like3 Module Regulates Ambient Temperature-Responsive Flowering via Flowering Locus T in Arabidopsis. Plant Physiol..

[149] Legris M., Klose C., Burgie E.S., Rojas C.C., Neme M., Hiltbrunner A., Wigge P.A., Schäfer E., Vierstra R.D., Casal J.J. (2016). Phytochrome B Integrates Light and Temperature Signals in Arabidopsis. Science.

[150] Jang K., Lee H.G., Jung S.J., Paek N.C., Seo P.J. (2015). The E3 Ubiquitin Ligase COP1 Regulates Thermosensory Flowering by Triggering GI Degradation in Arabidopsis. Sci. Rep..

[151] Luo X., He Y. (2020). Experiencing Winter for Spring Flowering: A Molecular Epigenetic Perspective on Vernalization. J. Integr. Plant Biol..

[152] Li Z., Jiang D., He Y. (2018). FRIGIDA Establishes a Local Chromosomal Environment for Flowering Locus C MRNA Production. Nat. Plants.

[153] Choi K., Kim J., Hwang H.J., Kim S., Park C., Kim S.Y., Lee I. (2011). The FRIGIDA Complex Activates Transcription of FLC, a Strong Flowering Repressor in Arabidopsis, by Recruiting Chromatin Modification Factors. Plant Cell.

[154] Ding L., Kim S.Y., Michaels S.D. (2013). FLOWERING LOCUS C EXPRESSOR Family Proteins Regulate FLOWERING LOCUS C Expression in Both Winter-Annual and Rapid-Cycling Arabidopsis. Plant Physiol..

[155] Crevillén P., Sonmez C., Wu Z., Dean C. (2013). A Gene Loop Containing the Floral Repressor FLC Is Disrupted in the Early Phase of Vernalization. EMBO J..

[156] Jégu T., Latrasse D., Delarue M., Hirt H., Domenichini S., Ariel F., Crespi M., Bergounioux C., Raynaud C., Benhamed M. (2014). The BAF60 Subunit of the SWI/SNF Chromatin-Remodeling Complex Directly Controls the Formation of a Gene Loop at FLOWERING LOCUS C in Arabidopsis. Plant Cell.

[157] Sun Q., Csorba T., Skourti-Stathaki K., Proudfoot N.J., Dean C. (2013). R-Loop Stabilization Represses Antisense Transcription at the Arabidopsis FLC Locus. Science.

[158] Csorba T., Questa J.I., Sun Q., Dean C. (2014). Antisense COOLAIR Mediates the Coordinated Switching of Chromatin States at FLC during Vernalization. Proc. Natl. Acad. Sci. USA.

[159] Kim D.H., Sung S. (2017). Vernalization-Triggered Intragenic Chromatin Loop Formation by Long Noncoding RNAs. Develop. Cell.

[160] Swiezewski S., Liu F., Magusin A., Dean C. (2009). Cold-Induced Silencing by Long Antisense Transcripts of an Arabidopsis Polycomb Target. Nature.

[161] Costa S., Dean C. (2019). Storing Memories: The Distinct Phases of Polycomb-Mediated Silencing of Arabidopsis FLC. Biochem. Soc. Trans..

[162] Yang H., Howard M., Dean C. (2014). Antagonistic Roles for H3K36me3 and H3K27me3 in the Cold-Induced Epigenetic Switch at Arabidopsis FLC. Curr. Biol..

[163] Qüesta J.I., Song J., Geraldo N., An H., Dean C. (2016). Arabidopsis Transcriptional Repressor VAL1 Triggers Polycomb Silencing at FLC during Vernalization. Science.

[164] Yuan W., Luo X., Li Z., Yang W., Wang Y., Liu R., Du J., He Y. (2016). A Cis Cold Memory Element and a Trans Epigenome Reader Mediate Polycomb Silencing of FLC by Vernalization in Arabidopsis. Nat. Genet..

[165] Zeng X., Gao Z., Jiang C., Yang Y., Liu R., He Y. (2020). HISTONE DEACETYLASE 9 Functions with Polycomb Silencing to Repress FLOWERING LOCUS C Expression. Plant Physiol..

[166] De Lucia F., Crevillen P., Jones A.M.E., Greb T., Dean C. (2008). A PHD-Polycomb Repressive Complex 2 Triggers the Epigenetic Silencing of FLC during Vernalization. Proc. Natl. Acad. Sci. USA.

[167] Yang H., Berry S., Olsson T.S.G., Hartley M., Howard M., Dean C. (2017). Distinct Phases of Polycomb Silencing to Hold Epigenetic Memory of Cold in Arabidopsis. Science.

[168] Kim D.H., Sung S. (2013). Coordination of the Vernalization Response through a VIN3 and FLC Gene Family Regulatory Network in Arabidopsis. Plant Cell.

[169] Kim D.H., Sung S. (2017). The Binding Specificity of the PHD-Finger Domain of VIN3 Moderates Vernalization Response. Plant Physiol..

[170] Zhao Y., Antoniou-Kourounioti R.L., Calder G., Dean C., Howard M. (2020). Temperature-Dependent Growth Contributes to Long-Term Cold Sensing. Nature.

[171] Heo J.B., Sung S. (2010). Vernalization-Mediated Epigenetic Silencing by a Long Intronic Noncoding RNA. Science.

[172] Coupland G. (2019). Flowering Locus c Isolation and Characterization: Two Articles That Opened Many Doors. Plant Cell.

[173] Xi Y., Park S.R., Kim D.H., Kim E.D., Sung S. (2020). Transcriptome and Epigenome Analyses of Vernalization in Arabidopsis Thaliana. Plant J..

[174] Rouse D.T., Sheldon C.C., Bagnall D.J., Peacock W.J., Dennis E.S. (2002). FLC, a Repressor of Flowering, Is Regulated by Genes in Different Inductive Pathways. Plant J..

[175] Michaels S.D., Ditta G., Gustafson-Brown C., Pelaz S., Yanofsky M., Amasino R.M. (2003). AGL24 Acts as a Promoter of Flowering in Arabidopsis and Is Positively Regulated by Vernalization. Plant J..

[176] Schönrock N., Bouveret R., Leroy O., Borghi L., Köhler C., Gruissem W., Hennig L. (2006). Polycomb-Group Proteins Repress the Floral Activator AGL19 in the FLC-Independent Vernalization Pathway. Genes Develop..

[177] Kim W., Latrasse D., Servet C., Zhou D.X. (2013). Arabidopsis Histone Deacetylase HDA9 Regulates Flowering Time through Repression of AGL19. Biochem. Biophys. Res. Commun..

[178] Kang M.J., Jin H.S., Noh Y.S., Noh B. (2015). Repression of Flowering under a Noninductive Photoperiod by the HDA9-AGL19-FT Module in Arabidopsis. New Phytol..

[179] Alexandre C.M., Hennig L. (2008). FLC or Not FLC: The Other Side of Vernalization. J. Exp. Bot..

[180] Torti S., Fornara F., Vincent C., Andrés F., Nordström K., Göbel U., Knoll D., Schoof H., Coupland G. (2012). Analysis of the Arabidopsis Shoot Meristem Transcriptome during Floral Transition Identifies Distinct Regulatory Patterns and a Leucine-Rich Repeat Protein That Promotes Flowering. Plant Cell.

[181] Yu H., Xu Y., Tan E.L., Kumar P.P. (2002). AGAMOUS-like 24, a Dosage-Dependent Mediator of the Flowering Signals. Proc. Natl. Acad. Sci. USA.

[182] Paul M.J., Primavesi L.F., Jhurreea D., Zhang Y. (2008). Trehalose Metabolism and Signaling. Annu. Rev. Plant Biol..

[183] Cho L.H., Pasriga R., Yoon J., Jeon J.S., An G. (2018). Roles of Sugars in Controlling Flowering Time. J. Plant Biol..

[184] Teotia S., Tang G. (2015). To Bloom or Not to Bloom: Role of Micrornas in Plant Flowering. Mol. Plant.

[185] Bao S., Hua C., Shen L., Yu H. (2020). New Insights into Gibberellin Signaling in Regulating Flowering in Arabidopsis. J. Integr. Plant Biol..

[186] Aukerman M.J., Sakai H. (2003). Regulation of Flowering Time and Floral Organ Identity by a MicroRNA and Its APETALA2-like target genes. Plant Cell.

[187] Jung J.H., Seo P.J., Kang S.K., Park C.M. (2011). MiR172 Signals Are Incorporated into the MiR156 Signaling Pathway at the SPL3/4/5 Genes in Arabidopsis Developmental Transitions. Plant Mol. Biol..

[188] Rhoades M.W., Reinhart B.J., Lim L.P., Burge C.B., Bartel B., Bartel D.P. (2002). Prediction of Plant MicroRNA Targets The Major Challenge in Determining MiRNA Functions. Cell.

[189] Cardon G., Höhmann S., Klein J., Nettesheim K., Saedler H., Huijser P. (1999). Molecular Characterisation of the Arabidopsis SBP-Box Genes. Gene.

[190] Yamaguchi A., Wu M.F., Yang L., Wu G., Poethig R.S., Wagner D. (2009). The MicroRNA-Regulated SBP-Box Transcription Factor SPL3 Is a Direct Upstream Activator of LEAFY, FRUITFULL, and APETALA1. Develop. Cell.

[191] Hyun Y., Richter R., Vincent C., Martinez-Gallegos R., Porri A., Coupland G. (2016). Multi-Layered Regulation of SPL15 and Cooperation with SOC1 Integrate Endogenous Flowering Pathways at the Arabidopsis Shoot Meristem. Develop. Cell.

[192] Xu M., Hu T., Zhao J., Park M.Y., Earley K.W., Wu G., Yang L., Poethig R.S. (2016). Developmental Functions of MiR156-Regulated SQUAMOSA PROMOTER BINDING PROTEIN-LIKE (SPL) Genes in Arabidopsis Thaliana. PLoS Genet..

[193] Yao T., Park B.S., Mao H.Z., Seo J.S., Ohama N., Li Y., Yu N., Mustafa N.F.B., Huang C.H., Chua N.H. (2019). Regulation of Flowering Time by SPL10/MED25 Module in Arabidopsis. New Phytol..

[194] Jung J.H., Lee H.J., Ryu J.Y., Park C.M. (2016). SPL3/4/5 Integrate Developmental Aging and Photoperiodic Signals into the FT-FD Module in Arabidopsis Flowering. Mol. Plant.

[195] Xie Y., Zhou Q., Zhao Y., Li Q., Liu Y., Ma M., Wang B., Shen R., Zheng Z., Wang H. (2020). FHY3 and FAR1 Integrate Light Signals with the MiR156-SPL Module-Mediated Aging Pathway to Regulate Arabidopsis Flowering. Mol. Plant.

[196] Ó’Maoiléidigh D.S., van Driel A.D., Singh A., Sang Q., Le Bec N., Vincent C., de Olalla E.B.G., Vayssières A., Romera Branchat M., Severing E. (2021). Systematic Analyses of the MIR172 Family Members of Arabidopsis Define Their Distinct Roles in Regulation of APETALA2 during Floral Transition. PLoS Biol..

[197] Lang A. (1957). The Effect of Gibberellin upon Flower Formation. Proc. Natl. Acad. Sci. USA.

[198] Eriksson S. (2006). GA4 Is the Active Gibberellin in the Regulation of LEAFY Transcription and Arabidopsis Floral Initiation. Plant Cell Online.

[199] Silverstone A.L., Jung H.S., Dill A., Kawaide H., Kamiya Y., Sun T.P. (2001). Repressing a Repressor: Gibberellin-Induced Rapid Reduction of the RGA Protein in Arabidopsis. Plant Cell.

[200] McGinnis K.M., Thomas S.G., Soule J.D., Strader L.C., Zale J.M., Sun T.P., Steber C.M. (2003). The Arabidopsis SLEEPY1 Gene Encodes a Putative F-Box Subunit of an SCF E3 Ubiquitin Ligase. Plant Cell.

[201] Peng J., Carol P., Richards D.E., King K.E., Cowling R.J., Murphy G.P., Harberd N.P. (1997). The Arabidopsis GAI Gene Defines a Signaling Pathway That Negatively Regulates Gibberellin Responses. Genes Develop..

[202] Silverstone A.L., Mak P.Y.A., Martínez E.C., Sun T.P. (1997). The New RGA Locus Encodes a Negative Regulator of Gibberellin Response in Arabidopsis Thaliana. Genetics.

[203] Dill A., Sun T.P. (2001). Synergistic Derepression of Gibberellin Signaling by Removing RGA and GAI Function in Arabidopsis Thaliana. Genetics.

[204] Tyler L., Thomas S.G., Hu J., Dill A., Alonso J.M., Ecker J.R., Sun T.P. (2004). Della Proteins and Gibberellin-Regulated Seed Germination and Floral Development in Arabidopsis. Plant Physiol..

[205] Thomas S.G., Blázquez M.A., Alabadí D. (2016). Della Proteins: Master Regulators of Gibberellin-Responsive Growth and Development. Annu. Plant Rev. Online.

[206] Wang H., Pan J., Li Y., Lou D., Hu Y., Yu D. (2016). The DELLA-CONSTANS Transcription Factor Cascade Integrates Gibberellic Acid and Photoperiod Signaling to Regulate Flowering. Plant Physiol..

[207] Xu F., Li T., Xu P.B., Li L., Du S.S., Lian H.L., Yang H.Q. (2016). DELLA Proteins Physically Interact with CONSTANS to Regulate Flowering under Long Days in Arabidopsis. FEBS Lett..

[208] Dill A., Thomas S.G., Hu J., Steber C.M., Sun T.P. (2004). The Arabidopsis F-Box Protein SLEEPY1 Targets Gibberellin Signaling Repressors for Gibberellin-Induced Degradation. Plant Cell.

[209] Nakajima M., Shimada A., Takashi Y., Kim Y.C., Park S.H., Ueguchi-Tanaka M., Suzuki H., Katoh E., Iuchi S., Kobayashi M. (2006). Identification and Characterization of Arabidopsis Gibberellin Receptors. Plant J..

[210] Murase K., Hirano Y., Sun T.P., Hakoshima T. (2008). Gibberellin-Induced DELLA Recognition by the Gibberellin Receptor GID1. Nature.

[211] Davière J., Achard P. (2013). Gibberellin Signaling in Plants. Development.

[212] Yu S., Galvao V.C., Zhang Y.-C., Horrer D., Zhang T.-Q., Hao Y.-H., Feng Y.-Q., Wang S., Schmid M., Wang J.-W. (2012). Gibberellin Regulates the Arabidopsis Floral Transition through MiR156-Targeted squamosa promoter binding-like Transcription Factors. Plant Cell.

[213] Fornara F., de Montaigu A., Coupland G. (2010). SnapShot: Control of Flowering in Arabidopsis. Cell.

[214] Wilson R.N., Heckman J.W., Somerville C.R. (1992). Gibberellin Is Required for Flowering in Arabidopsis Thaliana under Short Days. Plant Physiol..

[215] Sharma N., Xin R., Kim D.H., Sung S., Lange T., Huq E. (2016). NO FLOWERING IN SHORT DAY (NFL) Is a BHLH Transcription Factor That Promotes Flowering Specifically under Short-Day Conditions in Arabidopsis. Development.

[216] Li Y., Wang H., Li X., Liang G., Yu D. (2017). Two DELLA-Interacting Proteins BHLH48 and BHLH60 Regulate Flowering under Long-Day Conditions in Arabidopsis Thaliana. J. Exp. Bot..

[217] Bao S., Hua C., Huang G., Cheng P., Gong X., Shen L., Yu H. (2019). Molecular Basis of Natural Variation in Photoperiodic Flowering Responses. Develop. Cell.

[218] Jung J.H., Ju Y., Seo P.J., Lee J.H., Park C.M. (2012). The SOC1-SPL Module Integrates Photoperiod and Gibberellic Acid Signals to Control Flowering Time in Arabidopsis. Plant J..

[219] Yamaguchi N., Winter C.M., Wu M.F., Kanno Y., Yamaguchi A., Seo M., Wagner D. (2014). Gibberellin Acts Positively Then Negatively to Control Onset of Flower Formation in Arabidopsis. Science.

[220] Li M., An F., Li W., Ma M., Feng Y., Zhang X., Guo H. (2016). DELLA Proteins Interact with FLC to Repress Flowering Transition. J. Integr. Plant Biol..

[221] Richter R., Bastakis E., Schwechheimer C. (2013). Cross-Repressive Interactions between SOC1 and the GATAs GNC and GNL/CGA1 in the Control of Greening, Cold Tolerance, and Flowering Time in Arabidopsis. Plant Physiol..

[222] Yu Y., Liu Z., Wang L., Kim S.G., Seo P.J., Qiao M., Wang N., Li S., Cao X., Park C.M. (2016). WRKY71 Accelerates Flowering via the Direct Activation of FLOWERING LOCUS T and LEAFY in Arabidopsis Thaliana. Plant J..

[223] Zhang L., Chen L., Yu D. (2018). Transcription Factor WRKY75 Interacts with DELLA Proteins to Affect Flowering. Plant Physiol..

[224] Ma Z., Li W., Wang H., Yu D. (2020). WRKY Transcription Factors WRKY12 and WRKY13 Interact with SPL10 to Modulate Age-Mediated Flowering. J. Integr. Plant Biol..

[225] Li W., Wang H., Yu D. (2016). Arabidopsis WRKY Transcription Factors WRKY12 and WRKY13 Oppositely Regulate Flowering under Short-Day Conditions. Mol. Plant.

[226] Balanzà V., Martínez-Fernández I., Ferrándiz C. (2014). Sequential Action of FRUITFULL as a Modulator of the Activity of the Floral Regulators SVP and SOC1. J. Exp. Bot..

[227] Liu C., Chen H., Er H.L., Soo H.M., Kumar P.P., Han J.H., Liou Y.C., Yu H. (2008). Direct Interaction of AGL24 and SOC1 Integrates Flowering Signals in Arabidopsis. Development.

[228] Pérez-Ruiz R.V., García-Ponce B., Marsch-Martínez N., Ugartechea-Chirino Y., Villajuana-Bonequi M., De Folter S., Azpeitia E., Dávila-Velderrain J., Cruz-Sánchez D., Garay-Arroyo A. (2015). XAANTAL2 (AGL14) Is an Important Component of the Complex Gene Regulatory Network That Underlies Arabidopsis Shoot Apical Meristem Transitions. Mol. Plant.

[229] Lee J., Oh M., Park H., Lee I. (2008). SOC1 Translocated to the Nucleus by Interaction with AGL24 Directly Regulates LEAFY. Plant J..

[230] Gocal G.F.W., Sheldon C.C., Gubler F., Moritz T., Bagnall D.J., Macmillan C.P., Li S.F., Parish R.W., Dennis E.S., Weigel D. (2001). GAMYB-like Genes, Flowering, and Gibberellin Signaling in Arabidopsis. Plant Physiol..

[231] Bernier G., Havelange A., Houssa C., Petitjean A., Lejeune P. (1993). Physiological Signals That Induce Flowering. Plant Cell.

[232] Corbesier L., Lejeune P., Bernier G. (1998). The Role of Carbohydrates in the Induction of Flowering in Arabidopsis Thaliana: Comparison between the Wild Type and a Starchless Mutant. Planta.

[233] Ohto M.A., Onai K., Furukawa Y., Aoki E., Araki T., Nakamura K. (2001). Effects of Sugar on Vegetative Development and Floral Transition in Arabidopsis. Plant Physiol..

[234] Andrés F., Kinoshita A., Kalluri N., Fernández V., Falavigna V.S., Cruz T.M.D., Jang S., Chiba Y., Seo M., Mettler-Altmann T. (2020). The Sugar Transporter SWEET10 Acts Downstream of FLOWERING LOCUS T during Floral Transition of Arabidopsis Thaliana. BMC Plant Biol..

[235] Zhao H., Lin K., Ma L., Chen Q., Gan S., Li G. (2020). Arabidopsis NUCLEAR FACTOR Y A8 Inhibits the Juvenile-to-Adult Transition by Activating Transcription of MIR156s. J. Exp. Bot..

[236] Smaczniak C., Immink R.G.H., Muiño J.M., Blanvillain R., Busscher M., Busscher-Lange J., Dinh Q.D., Liu S., Westphal A.H., Boeren S. (2012). Characterization of MADS-Domain Transcription Factor Complexes in Arabidopsis Flower Development. Proc. Natl. Acad. Sci. USA.

[237] Searle I., Coupland G. (2004). Induction of Flowering by Seasonal Changes in Photoperiod. EMBO J..

[238] An H., Roussot C., Suárez-López P., Corbesier L., Vincent C., Piñeiro M., Hepworth S., Mouradov A., Justin S., Turnbull C. (2004). CONSTANS Acts in the Phloem to Regulate a Systemic Signal That Induces Photoperiodic Flowering of Arabidopsis. Development.

[239] Imaizumi T., Tran H.G., Swartz T.E., Briggs W.R., Kay S.A. (2003). FKF1 Is Essential for Photoperiodic-Specific Light Signalling in Arabidopsis. Nature.

[240] Imaizumi T., Schultz T.F., Harmon F.G., Ho L.A., Kay S.A. (2005). Plant Science: FKF1 F-Box Protein Mediates Cyclic Degradation of a Repressor of CONSTANS in Arabidopsis. Science.

[241] Fornara F., Panigrahi K.C.S., Gissot L., Sauerbrunn N., Rühl M., Jarillo J.A., Coupland G. (2009). Arabidopsis DOF Transcription Factors Act Redundantly to Reduce CONSTANS Expression and Are Essential for a Photoperiodic Flowering Response. Develop. Cell.

[242] Song Y.H., Smith R.W., To B.J., Millar A.J., Imaizumi T. (2012). FKF1 Conveys Timing Information for CONSTANS Stabilization in Photoperiodic Flowering. Science.

[243] Kim W.Y., Fujiwara S., Suh S.S., Kim J., Kim Y., Han L., David K., Putterill J., Nam H.G., Somers D.E. (2007). ZEITLUPE Is a Circadian Photoreceptor Stabilized by GIGANTEA in Blue Light. Nature.

[244] Park D.H., Somers D.E., Kim Y.S., Choy Y.H., Lim H.K., Soh M.S., Kim H.J., Kay S.A., Nam H.G. (1999). Control of Circadian Rhythms and Photoperiodic Flowering by the Arabidopsis GIGANTEA Gene. Science.

[245] Fowler S., Lee K., Onouchi H., Samach A., Richardson K., Morris B., Coupland G., Putterill J. (1999). GIGANTEA: A Circadian Clock-Controlled Gene That Regulates Photoperiodic Flowering in Arabidopsis and Encodes a Protein with Several Possible Membrane-Spanning Domains. EMBO J..

[246] Kubota A., Ito S., Shim J.S., Johnson R.S., Song Y.H., Breton G., Goralogia G.S., Kwon M.S., Laboy Cintrón D., Koyama T. (2017). TCP4-Dependent Induction of CONSTANS Transcription Requires GIGANTEA in Photoperiodic Flowering in Arabidopsis. PLoS Gene..

[247] Ito S., Song Y.H., Josephson-Day A.R., Miller R.J., Breton G., Olmstead R.G., Imaizumi T. (2012). FLOWERING BHLH Transcriptional Activators Control Expression of the Photoperiodic Flowering Regulator CONSTANS in Arabidopsis. Proc. Natl. Acad. Sci. USA.

[248] Liu L.J., Zhang Y.C., Li Q.H., Sang Y., Mao J., Lian H.L., Wang L., Yang H.Q. (2008). COP1-Mediated Ubiquitination of CONSTANS Is Implicated in Cryptochrome Regulation of Flowering in Arabidopsis. Plant Cell.

[249] Shim J.S., Kubota A., Imaizumi T. (2017). Circadian Clock and Photoperiodic Flowering in Arabidopsis: CONSTANS Is a Hub for Signal Integration. Plant Physiol..

[250] Laubinger S., Marchal V., Le Gourrierec J., Wenkel S., Adrian J., Jang S., Kulajta C., Braun H., Coupland G., Hoecker U. (2006). Arabidopsis SPA Proteins Regulate Photoperiodic Flowering and Interact with the Floral Inducer CONSTANS to Regulate Its Stability. Development.

[251] Jang S., Marchal V., Panigrahi K.C.S., Wenkel S., Soppe W., Deng X., Coupland G. (2008). Arabidopsis COP1 Shapes the Temporal Pattern of CO Accumulation Conferring a Photoperiodic Flowering Response. EMBO J..

[252] Yanovsky M.J., Kay S.A. (2002). Molecular Basis of Seasonal Time Measurement in Arabidopsis. Nature.

[253] Valverde F., Mouradov A., Soppe W., Ravenscroft D., Samach A., Coupland G. (2004). Photoreceptor Regulation of CONSTANS Protein in Photoperiodic Flowering. Science.

[254] Zuo Z., Liu H., Liu B., Liu X., Lin C. (2011). Blue Light-Dependent Interaction of CRY2 with SPA1 Regulates COP1 Activity and Floral Initiation in Arabidopsis. Curr. Biol..

[255] Sanchez-Bermejo E., Zhu W., Tasset C., Eimer H., Sureshkumar S., Singh R., Sundaramoorthi V., Colling L., Balasubramanian S. (2015). Genetic Architecture of Natural Variation in Thermal Responses of Arabidopsis. Plant Physiol..

[256] El-assal S.E., Alonso-blanco C., Peeters A.J.M., Raz V., Koornneef M. (2001). A QTL for Flowering Time in Arabidopsis Reveals a Novel Allele of CRY2. Nat. Genet..

[257] Lazaro A., Valverde F., Piñeiro M., Jarillo J.A. (2012). The Arabidopsis E3 Ubiquitin Ligase HOS1 Negatively Regulates CONSTANS Abundance in the Photoperiodic Control of Flowering. Plant Cell.

[258] Lazaro A., Mouriz A., Piñeiro M., Jarillo J.A. (2015). Red Light-Mediated Degradation of Constans by the E3 Ubiquitin Ligase Hos1 Regulates Photoperiodic Flowering in Arabidopsis. Plant Cell.

[259] Endo M., Tanigawa Y., Murakami T., Araki T., Nagatani A. (2013). Phytochrome-Dependent Late-Flowering Accelerates Flowering through Physical Interactions with Phytochrome B and CONSTANS. Proc. Natl. Acad. Sci. USA.

[260] Hwang D.Y., Park S., Lee S., Lee S.S., Imaizumi T., Song Y.H. (2019). GIGANTEA Regulates the Timing Stabilization of CONSTANS by Altering the Interaction between FKF1 and ZEITLUPE. Mol. Cells.

[261] Song Y.H., Estrada D.A., Johnson R.S., Kim S.K., Lee S.Y., MacCoss M.J., Imaizumi T. (2014). Distinct Roles of FKF1, GIGANTEA, and ZEITLUPE Proteins in the Regulation of Constans Stability in Arabidopsis Photoperiodic Flowering. Proc. Natl. Acad. Sci. USA.

[262] Yan J., Li X., Zeng B., Zhong M., Yang J., Yang P., Li X., He C., Lin J., Liu X. (2020). FKF1 F-Box Protein Promotes Flowering in Part by Negatively Regulating DELLA Protein Stability under Long-Day Photoperiod in Arabidopsis. J. Integr. Plant Biol..

[263] Zhang B., Wang L., Zeng L., Zhang C., Ma H. (2015). Arabidopsis TOE Proteins Convey a Photoperiodic Signal to Antagonize CONSTANS and Regulate Flowering Time. Genes Develop..

[264] Tiwari S.B., Shen Y., Chang H.C., Hou Y., Harris A., Ma S.F., McPartland M., Hymus G.J., Adam L., Marion C. (2010). The Flowering Time Regulator CONSTANS Is Recruited to the FLOWERING LOCUS T Promoter via a Unique Cis-Element. New Phytol..

[265] Corbesier L., Vincent C., Jang S., Fornara F., Fan Q., Searle I., Giakountis A., Farrona S., Gissot L., Turnbull C. (2007). FT Protein Movement Contributes to Long-Distance Signaling in Floral Induction of Arabidopsis. Science.

[266] Jaeger K.E., Wigge P.A. (2007). FT Protein Acts as a Long-Range Signal in Arabidopsis. Curr. Biol..

[267] Pin P.A., Nilsson O. (2012). The Multifaceted Roles of FLOWERING LOCUS T in Plant Development. Plant Cell Environ..

[268] Liu L., Liu C., Hou X., Xi W., Shen L., Tao Z., Wang Y., Yu H. (2012). FTIP1 Is an Essential Regulator Required for Florigen Transport. PLoS Biol..

[269] Zhu Y., Liu L., Shen L., Yu H. (2016). NaKR1 Regulates Long-Distance Movement of FLOWERING LOCUS T in Arabidopsis. Nat. Plants.

[270] Kardailsky I., Shukla V.K., Ahn J.H., Dagenais N., Christensen S.K., Nguyen J.T., Chory J., Harrison M.J., Weigel D. (1999). Activation Tagging of the Floral Inducer FT. Science.

[271] Kobayashi Y., Kaya H., Goto K., Iwabuchi M. (1999). A Pair of Related Genes with Antagonistic Roles in Mediating Flowering Signals. Science.

[272] Yoo S.Y., Kardailsky I., Lee J.S., Weigel D., Ahn J.H. (2004). Acceleration of Flowering by Overexpression of MFT (MOTHER OF FT AND TFL1). Mol. Cells.

[273] Yamaguchi A., Kobayashi Y., Goto K., Abe M., Araki T. (2005). TWIN SISTER of FT (TSF) Acts as a Floral Pathway Integrator Redundantly with FT. Plant Cell Physiol..

[274] Taoka K.I., Ohki I., Tsuji H., Furuita K., Hayashi K., Yanase T., Yamaguchi M., Nakashima C., Purwestri Y.A., Tamaki S. (2011). 14-3-3 Proteins Act as Intracellular Receptors for Rice Hd3a Florigen. Nature.

[275] Collani S., Neumann M., Yant L., Schmid M. (2019). FT Modulates Genome-Wide DNA-Binding of the BZIP Transcription Factor FD. Plant Physiol..

[276] Abe M., Kobayashi Y., Yamamoto S., Daimon Y., Yamaguchi A., Ikeda Y., Ichinoki H., Notaguchi M., Goto K., Araki T. (2005). FD, a BZIP Protein Mediating Signals from the Floral Pathway Integrator FT at the Shoot Apex. Science.

[277] Wigge P.A., Kim M.C., Jaeger K.E., Busch W., Schmid M., Lohmann J.U., Weigel D. (2005). Integration of Spatial and Temporal Information during Floral Induction in Arabidopsis. Science.

[278] Abe M., Kosaka S., Shibuta M., Nagata K., Uemura T., Nakano A., Kaya H. (2019). Transient Activity of the Florigen Complex during the Floral Transition in Arabidopsis Thaliana. Development.

[279] Kim S.J., Hong S.M.M., Yoo S.J.J., Moon S., Jung H.S.S., Ahn J.H.H. (2016). Post-Translational Regulation of FLOWERING LOCUS T Protein in Arabidopsis. Mol. Plant.

[280] Yoo S.K., Chung K.S., Kim J., Lee J.H., Hong M.S., Yoo S.J., Yoo S.Y., Jong S.L., Ahn J.H. (2005). CONSTANS Activates SUPPRESSOR OF OVEREXPRESSION OF CONSTANS 1 through FLOWERING LOCUS T to Promote Flowering in Arabidopsis. Plant Physiol..

[281] Hisamatsu T., King R.W. (2008). The Nature of Floral Signals in Arabidopsis. II. Roles for FLOWERING LOCUS T and Gibberellin. J. Exp. Bot..

[282] Han P., García-Ponce B., Fonseca-Salazar G., Alvarez-Buylla E.R., Yu H. (2008). AGAMOUS-LIKE 17, a Novel Flowering Promoter, Acts in a FT-Independent Photoperiod Pathway. Plant J..

[283] Wagner D., Sablowski R.W.M., Meyerowitz E.M. (1999). Transcriptional Activation of APETALA1 by LEAFY. Science.

[284] William D.A., Su Y., Smith M.R., Lu M., Baldwin D.A., Wagner D. (2004). Genomic Identification of Direct Target Genes of LEAFY. Proc. Natl. Acad. Sci. USA.

[285] Kaufmann K., Wellmer F., Muiñ J.M., Ferner T., Wuest S.E., Kumar V., Serrano-Mislata A., Madueño F., Kraiewski P., Meyerowitz E.M. (2010). Orchestration of Floral Initiation by APETALA1. Science.

[286] Benlloch R., Kim M.C., Sayou C., Thévenon E., Parcy F., Nilsson O. (2011). Integrating Long-Day Flowering Signals: A LEAFY Binding Site Is Essential for Proper Photoperiodic Activation of APETALA1. Plant J..

[287] Kumar S.V., Lucyshyn D., Jaeger K.E., Alós E., Alvey E., Harberd N.P., Wigge P.A. (2012). Transcription Factor PIF4 Controls the Thermosensory Activation of Flowering. Nature.

[288] Thines B.C., Youn Y., Duarte M.I., Harmon F.G. (2014). The Time of Day Effects of Warm Temperature on Flowering Time Involve PIF4 and PIF5. J. Exp. Bot..

[289] Galvāo V.C., Fiorucci A.S., Trevisan M., Franco-Zorilla J.M., Goyal A., Schmid-Siegert E., Solano R., Fankhauser C. (2019). PIF Transcription Factors Link a Neighbor Threat Cue to Accelerated Reproduction in Arabidopsis. Nat. Commun..

[290] Lorrain S., Allen T., Duek P.D., Whitelam G.C., Fankhauser C. (2008). Phytochrome-Mediated Inhibition of Shade Avoidance Involves Degradation of Growth-Promoting BHLH Transcription Factors. Plant J..

[291] Susila H., Nasim Z., Ahn J.H. (2018). Ambient Temperature-Responsive Mechanisms Coordinate Regulation of Flowering Time. Int. J. Mol. Sci..

[292] Fernández V., Takahashi Y., Le Gourrierec J., Coupland G. (2016). Photoperiodic and Thermosensory Pathways Interact through CONSTANS to Promote Flowering at High Temperature under Short Days. Plant J..

[293] Edwards K.D., Anderson P.E., Hall A., Salathia N.S., Locke J.C.W., Lynn J.R., Straume M., Smith J.Q., Millar A.J. (2006). FLOWERING LOCUS C Mediates Natural Variation in the High-Temperature Response of the Arabidopsis Circadian Clock. Plant Cell.

[294] Yamaguchi A., Abe M. (2012). Regulation of Reproductive Development by Non-Coding RNA in Arabidopsis: To Flower or Not to Flower. J. Plant Res..

[295] Romanowski A., Yanovsky M.J. (2015). Circadian Rhythms and Post-Transcriptional Regulation in Higher Plants. Front. Plant Sci..

[296] Zhang X., Guo H. (2017). MRNA Decay in Plants: Both Quantity and Quality Matter. Curr. Opin. Plant Biol..

[297] Seo P.J., Park M.J., Park C.M. (2013). Alternative Splicing of Transcription Factors in Plant Responses to Low Temperature Stress: Mechanisms and Functions. Planta.

[298] Capovilla G., Pajoro A., Immink R.G.H., Schmid M. (2015). Role of Alternative Pre-MRNA Splicing in Temperature Signaling. Curr. Opin. Plant Biol..

[299] Verhage L., Severing E.I., Bucher J., Lammers M., Busscher-Lange J., Bonnema G., Rodenburg N., Proveniers M.C.G., Angenent G.C., Immink R.G.H. (2017). Splicing-Related Genes Are Alternatively Spliced upon Changes in Ambient Temperatures in Plants. PLoS ONE.

[300] Wang Y.Y., Xiong F., Ren Q.P., Wang X.L. (2020). Regulation of Flowering Transition by Alternative Splicing: The Role of the U2 Auxiliary Factor. J. Exp. Bot..

[301] Bartok O., Kyriacou C.P., Levine J., Sehgal A., Kadener S. (2013). Adaptation of Molecular Circadian Clockwork to Environmental Changes: A Role for Alternative Splicing and MiRNAs. Proc. R. Soc. B Biol. Sci..

[302] Grandi V., Gregis V., Kater M.M. (2012). Uncovering Genetic and Molecular Interactions among Floral Meristem Identity Genes in Arabidopsis Thaliana. Plant J..

[303] Moyroud E., Minguet E.G., Ott F., Yant L., Posé D., Monniaux M., Blanchet S., Bastien O., Thévenon E., Weigel D. (2011). Prediction of Regulatory Interactions from Genome Sequences Using a Biophysical Model for the Arabidopsis LEAFY Transcription Factor. Plant Cell.

[304] Ye L., Wang B., Zhang W., Shan H., Kong H. (2016). Gains and Losses of Cis-Regulatory Elements Led to Divergence of Thearabidopsis APETALA1 and CAULIFLOWER Duplicate Genes in the Time, Space, and Level of Expression and Regulation of One Paralog by the Other. Plant Physiol..

[305] Wang J.W., Czech B., Weigel D. (2009). MiR156-Regulated SPL Transcription Factors Define an Endogenous Flowering Pathway in Arabidopsis Thaliana. Cell.

[306] Honma T., Goto K. (2001). Complexes of MADS-Box Proteins Are Suficient to Convert Leaves into Floral Organs. Nature.

[307] Yan W., Chen D., Kaufmann K. (2016). Molecular Mechanisms of Floral Organ Specification by MADS Domain Proteins. Curr. Opin. Plant Biol..

[308] Pajoro A., Verhage L., Immink R.G.H. (2016). Plasticity versus Adaptation of Ambient-Temperature Flowering Response. Trends Plant Sci..

[309] Li P., Filiault D., Box M.S., Kerdaffrec E., van Oosterhout C., Wilczek A.M., Schmitt J., McMullan M., Bergelson J., Nordborg M. (2014). Multiple FLC Haplotypes Defined by Independent Cisregulatory Variation Underpin Life History Diversity in Arabidopsis Thaliana. Genes Develop..

[310] Suter L., Rüegg M., Zemp N., Hennig L., Widmer A. (2014). Gene Regulatory Variation Mediates Flowering Responses to Vernalization along an Altitudinal Gradient in Arabidopsis. Plant Physiol..

[311] Hayama R., Coupland G. (2004). The Molecular Basis of Diversity in the Photoperiodic Flowering Responses of Arabidopsis and Rice. Plant Physiol..

[312] Higgins J.A., Bailey P.C., Laurie D.A. (2010). Comparative Genomics of Flowering Time Pathways Using Brachypodium Distachyon as a Model for the Temperate Grasses. PLoS ONE.

[313] Haspolat E., Huard B., Angelova M. (2019). Deterministic and Stochastic Models of Arabidopsis Thaliana Flowering. Bull. Math. Biol..

[314] Valentim F.L., Van Mourik S., Posé D., Kim M.C., Schmid M., Van Ham R.C.H.J., Busscher M., Sanchez-Perez G.F., Molenaar J., Angenent G.C. (2015). A Quantitative and Dynamic Model of the Arabidopsis Flowering Time Gene Regulatory Network. PLoS ONE.

[315] Jaeger K.E., Pullen N., Lamzin S., Morris R.J., Wigge P.A. (2013). Interlocking Feedback Loops Govern the Dynamic Behavior of the Floral Transition in Arabidopsis. Plant Cell.

